# Head Down Tilt 15° to Increase Collateral Flow in Acute Ischemic Stroke: Rationale and Study Protocol of a Multicenter, Randomized, Proof-of-Concept, Phase 2a/b Trial in Patients Treated With Mechanical Thrombectomy (DOWN-SUITE)

**DOI:** 10.1161/SVIN.125.002221

**Published:** 2026-02-04

**Authors:** Francesco Andrea Pedrazzini, Lorenzo Piergallini, Susanna Diamanti, Enrico Fainardi, Sergio Lucio Vinci, Caterina Sozzi, Matteo Farè, Emanuela Rossi, Francesca Graziano, Francesca Poggetti, Gabriele Mainini, Angela Giglio, Andrea Magi, Giulia Pederzoli, Agnese Anzani, Elisabetta De Bernardi, Valeria Cerina, Tae-Hee Cho, Fabien Chauveau, Davide Carone, Gianpaolo Basso, Giuseppe Citerio, Cristina Sarti, Nicola Limbucci, Francesco Janes, Carmela Casella, Antonio Toscano, Simona Sacco, Danilo Toni, Paolo Remida, Carlo Ferrarese, Simone Beretta

**Affiliations:** Department of Medicine and Surgery and Milan Center for Neuroscience (NeuroMI) (F.A.P., S.D., C.F., S.B.), University of Milano-Bicocca, Monza, Italy.; Department of Medicine and Surgery (C. Sozzi, M.F., E.R., F.P., G.M., A.G., A.M., G.P., A.A., E.D.B., V.C., G.B., G.C.), University of Milano-Bicocca, Monza, Italy.; Department of Neuroscience (L.P., S.D., G.C., P.R., C.F., S.B.), Fondazione IRCCS San Gerardo dei Tintori, Monza, Italy.; Biostatistics and Clinical Epidemiology (F.G.), Fondazione IRCCS San Gerardo dei Tintori, Monza, Italy.; Neuroradiology Unit, Department of Experimental and Clinical Biomedical Sciences, Careggi University Hospital (E.F.), University of Florence, Italy.; Neuroscience Section, NEUROFARBA Department (C. Sarti), University of Florence, Italy.; Neuroradiology Unit, University of Messina, Italy (S.L.V.).; Department of Vascular Neurology, Hospices Civils de Lyon, France (T.-H.C.).; Lyon Neuroscience Research Center, CNRS, INSERM, University of Lyon 1, France (F.C.).; Acute Vascular Imaging Centre, Radcliffe Department of Medicine, University of Oxford, United Kingdom (D.C.).; Neuroradiology Unit, Azienda Ospedaliero-Universitaria Careggi, Firenze, Italy (N.L.).; Department of Medicine, University of Udine and Clinical Neurology, Udine University Hospital, Italy (F.J.).; UOSD Stroke Unit, AOU G. Martino, Messina, Italy (C.C., A.T.).; Department of Biotechnological and Applied Clinical Sciences, University of L’Aquila, Italy (S.S.).; Department of Human Neurosciences, University of Rome La Sapienza, Italy (D.T.).

**Keywords:** feasibility studies, head down tilt, ischemic stroke, Italy, thrombectomy

## Abstract

**BACKGROUND:**

Collateral blood flow is a critical determinant of successful recanalization in acute ischemic stroke caused by large vessel occlusion. Head down tilt −15° (HDT15), similar to Trendelenburg positioning, is a simple, low-cost positional therapy that may augment cerebral collateral blood flow and penumbral survival. The aim of the study is to assess the safety, feasibility, and efficacy of HDT15 in improving cerebral collateral circulation and clinical outcomes in patients with large vessel occlusion–acute ischemic stroke treated with mechanical thrombectomy (MT).

**METHODS:**

The DOWN-SUITE trial (Head Down Tilt 15° to Increase Collateral Flow in Acute Ischemic Stroke) is a multicenter, randomized, open-label, phase 2a/b clinical trial with blinded outcome assessment, conducted across 7 Italian stroke centers. A total of 118 patients with acute ischemic stroke due to M1 segment middle cerebral artery occlusion will be randomized 1:1 in the emergency department to receive HDT15 or standard positioning (head-of-bed 0° to +30°) before and during MT.

**RESULTS:**

The primary end point is good collateral status (American Society of Interventional and Therapeutic Neuroradiology/Society of Interventional Radiology grade 3–4), assessed on the first angiographic sequence during MT by a blinded imaging core laboratory. Secondary end points include feasibility (proportion maintaining HDT15, admission-to-MT time), safety (symptomatic intracranial hemorrhage, pneumonia, vomiting, neurological deterioration, vital signs), and efficacy (neurological improvement before MT, at 24 hours, and at 7 days or discharge, modified Rankin Scale score at 90 days).

**CONCLUSIONS:**

The DOWN-SUITE trial will provide evidence on the acute cerebrovascular effect of HDT15 in large vessel occlusion–acute ischemic stroke, potentially establishing a cost-effective, practice-changing intervention to improve collaterals for global stroke care.

**REGISTRATION:**

URL: https://www.clinicaltrials.gov; Unique identifier: NCT06297863.

## Graphical abstract

**Figure:**
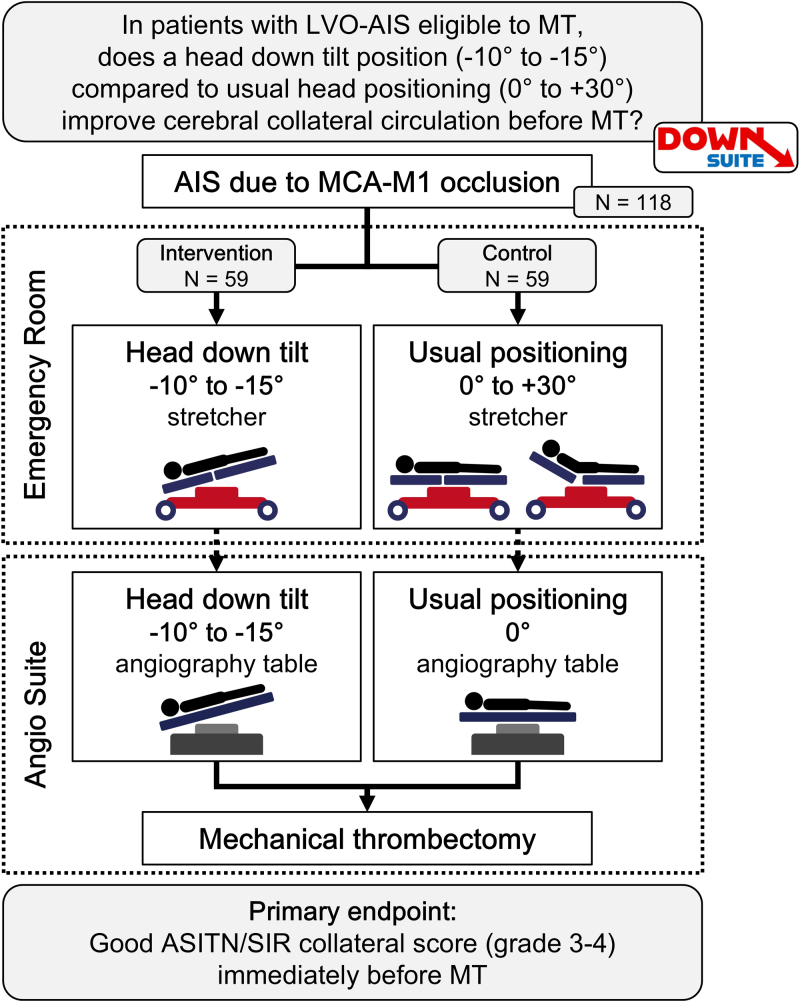

**CLINICAL PERSPECTIVE**

**What Is New?**

- The DOWN-SUITE trial (Head Down Tilt 15° to Increase Collateral Flow in Acute Ischemic Stroke) introduces the first multicenter, randomized, phase 2a/b study evaluating head down tilt −15° as a simple positional therapy to enhance leptomeningeal collateral flow, assessed via blinded angiographic grading, in patients with acute ischemic stroke with middle cerebral artery–M1 occlusion undergoing mechanical thrombectomy.

**What Are the Clinical Implications?**

- If head down tilt −15° proves safe and effective in improving collateral circulation, it could extend penumbral survival and improve thrombectomy outcomes, offering a low-cost, easily implementable intervention for hyperacute stroke care worldwide.
- Positive results would pave the groundwork for larger randomized controlled trials in broader stroke populations and diverse settings, potentially integrating head down tilt −15° into international stroke management guidelines to reduce disability globally.

Reperfusion therapies for acute ischemic stroke (AIS), including intravenous thrombolysis and mechanical thrombectomy (MT), are highly effective and have become the standard of care.^1^ However, their benefit depends on the subject-specific time window of salvageable ischemic brain tissue (penumbra), whose evolution to irreversible infarction depends on residual blood flow provided by cerebral collaterals.^2^ Despite recanalization, over up to 1 in 2 patients remain disabled, with the degree of collateral blood flow as a key predictor of successful versus futile recanalization.^3–5^ Developing a collateral therapeutic to enhance penumbra survival and improve stroke therapy efficacy is a major objective in stroke research. Several strategies have been proposed to boost collaterals in AIS, but none have been currently validated.^6–10^

Head down tilt −15° (HDT15) is a positional therapy consisting of tilting the supine patient by 15°, with the head lower than the body, similar to Trendelenburg positioning (Figure 1A). This approach aims to gravitationally divert blood flow from the lower body towards the head, potentially increasing cerebral blood flow in the penumbra.^11,12^ Preclinical studies in rodent models of middle cerebral artery (MCA) occlusion followed by recanalization showed that HDT15 increased cerebral blood flow in the ischemic hemisphere, reducing infarct volume and improving functional outcome.^13–15^

**Figure 1.**
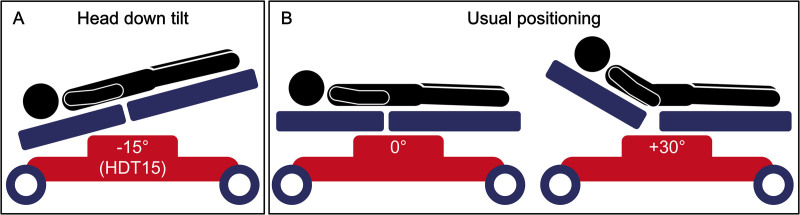
**Head down tilt −15° (HDT15) and usual positioning in patients with acute ischemic stroke during the hyperacute phase. A**, HDT15 involves tilting the supine patient 15° with the head lower than the body, similar to Trendelenburg positioning. **B**, Usual positioning involves head-of-bed angles ranging from 0° (**left**) to +30° (**right**).

There is no consensus on the optimal head position for patients with AIS, with Western countries often favoring head-of-bed (HOB) elevation at +30° or, less commonly, flat positioning at 0° (Figure 1B and 1C).^16^ The HeadPoST trial (Head Position in Stroke Trial), a cluster-randomized study of over 11 000 patients with acute stroke (85% ischemic), found no efficacy or safety differences between lying-flat (HOB at 0°) and sitting-up (HOB≥30°) positions maintained for 24 hours. However, the HeadPoST trial primarily targeted patients with mild symptoms without a large vessel occlusion (LVO), who were randomized beyond the usual time window of reperfusion therapies.^17^ The ZODIAC trial (Zero Degree Head Positioning in Hyperacute Large Artery Ischemic Stroke) showed HOB at 0° versus +30° safely reduced neurological deterioration in patients with LVO-AIS pre-MT, supporting a beneficial and rapid effect of lower HOB positioning in this clinical setting.^18^ Considering angles lower than 0°, an observational study from the prethrombectomy era suggested that HDT (0° to −15°) improved neurological outcomes in patients with AIS with LVO without increasing serious adverse events, compared with standard positioning (0° to +30°).^19^ Another trial reported better long-term outcomes with −20° positioning versus flat positioning, but it was limited to patients ineligible for recanalization, mainly with large-artery atherosclerotic or hemodynamic cause.^20^ Although these studies raised no safety concerns for HDT in AIS, efficacy of head positioning below 0° in patients undergoing MT remains unproven.

Given its simplicity, low cost, and feasibility, HDT15 is a promising collateral therapeutic candidate to prolong penumbral survival and enhance the clinical benefit of reperfusion therapies, ultimately reducing disability. We hypothesize that HDT15, applied in patients with AIS with LVO, will improve collateral circulation, extend penumbral survival, and enhance MT outcomes compared with standard positioning (0° to +30°). The DOWN-SUITE trial (Head Down Tilt 15° to Increase Collateral Flow in Acute Ischemic Stroke) aims to provide proof-of-concept evidence that head down tilt −15° (HDT15, range −10° to −15°) enhances leptomeningeal collateral flow in patients with AIS with MCA-M1 occlusion undergoing MT, measured by digital subtraction angiography. This is a critical mechanistic gap unaddressed by prior studies, which has yet to be examined. The control arm (0° to +30°) reflects standard clinical practice variability, reflecting current clinical practice variability due to the absence of guideline recommendations.

## Methods

### Data Availability Statement

The full-length study protocol and the Standard Protocol Items: Recommendations for Interventional Trials checklist are available as Supplemental Material.

### Study Design

**Figure 2.**
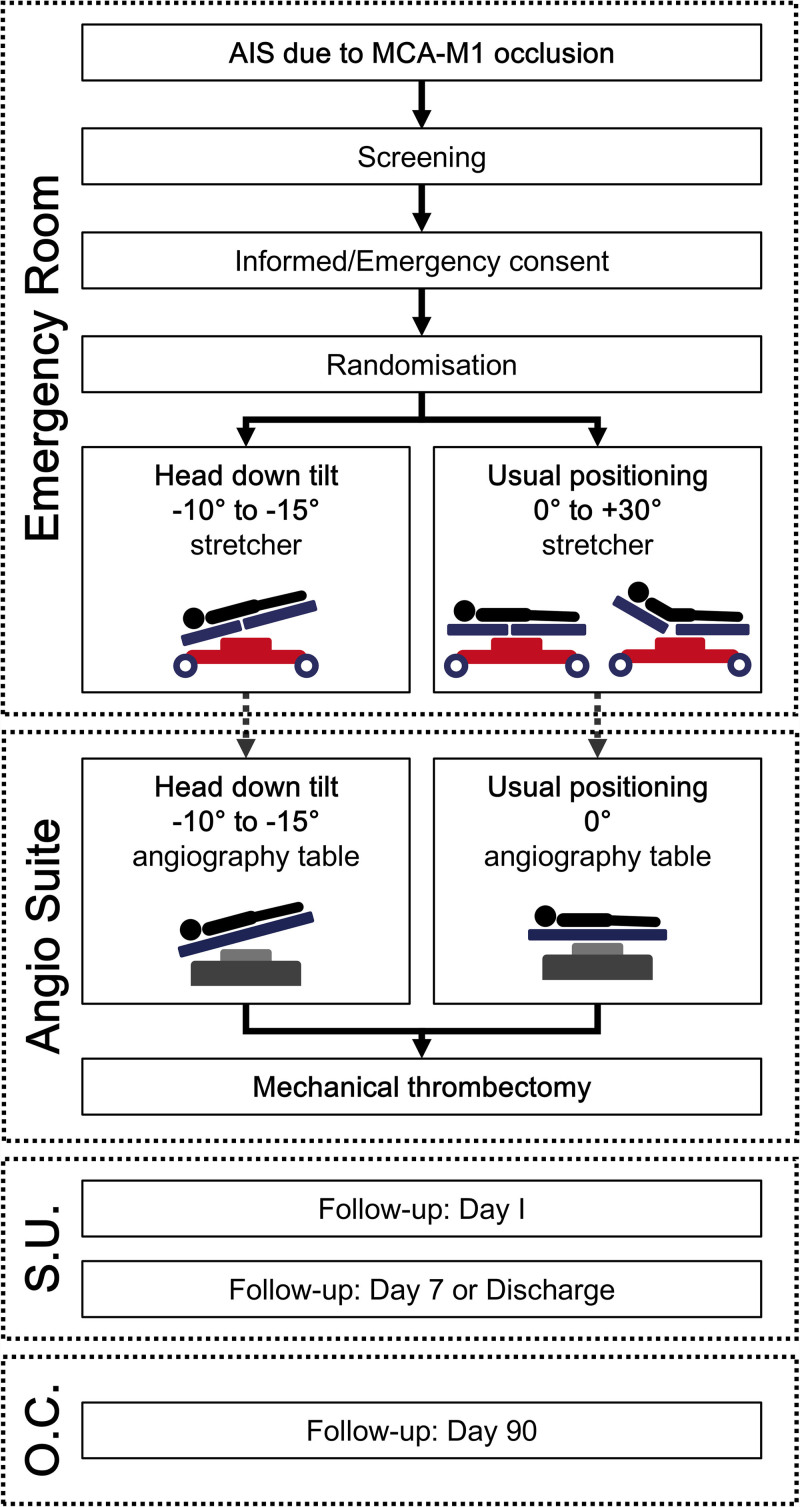
**Study flow chart.** AIS indicates acute ischemic stroke; MCA-M1, middle cerebral artery, M1 segment; O.C., outpatient clinic; and S.U., stroke unit.

The DOWN-SUITE is a multicenter, proof-of-concept, randomized, controlled, open-label, phase 2a/b clinical trial with blinded outcome assessment. It compares cerebral collateral status in patients with AIS due to MCA occlusion (M1 segment) treated with in-hospital application of HDT15 versus usual positioning (0° to +30°) before and during MT. Due to the nature of the intervention, double-blinding is not feasible; however, a blinded central imaging core laboratory, unaware of treatment assignments, will assess all imaging outcomes, including the primary efficacy end point. The trial flow chart is presented in Figure 2.

The trial adheres to the Declaration of Helsinki, Good Clinical Practice guidelines, and ethical approval has been obtained by Comitato Etico Lombardia 3 on June 19, 2024 (ID 4944_19.06.2024_M) and the local ethical committees of all the participating centers. The study protocol accords with the Standard Protocol Items: Recommendations for Interventional Trials guidelines.^21^

The study is funded by the Italian Ministry of University and Research, which has no role in the design or conduct of the trial. Patient enrollment began in March 2025.

### Patient Population

This trial includes adult patients with AIS due to isolated left or right MCA occlusion (M1 segment) who are eligible for MT, with or without intravenous thrombolysis. Patients will be excluded if they have a high risk of aspiration pneumonia, suspected elevated intracranial pressure, a history of glaucoma, or conditions predisposing to cardiopulmonary distress. The trial will be conducted across 7 Italian academic hospitals, each with a comprehensive high-volume stroke center. Inclusion and exclusion criteria are provided in Table 1, whereas baseline assessments and study procedures are outlined in Table 2.

**Table 1.**
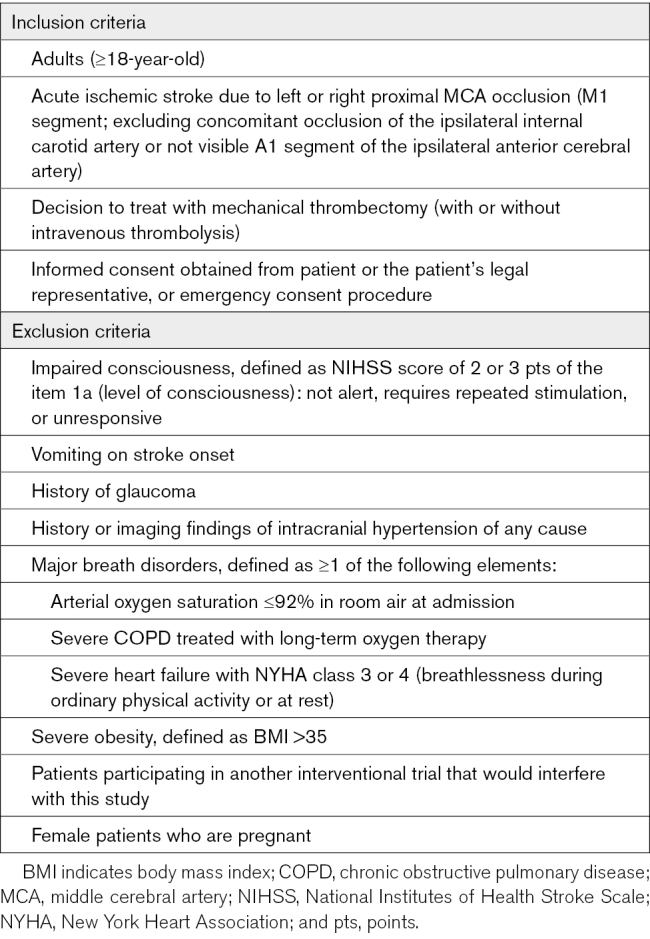
Inclusion and Exclusion Criteria

**Table 2.**
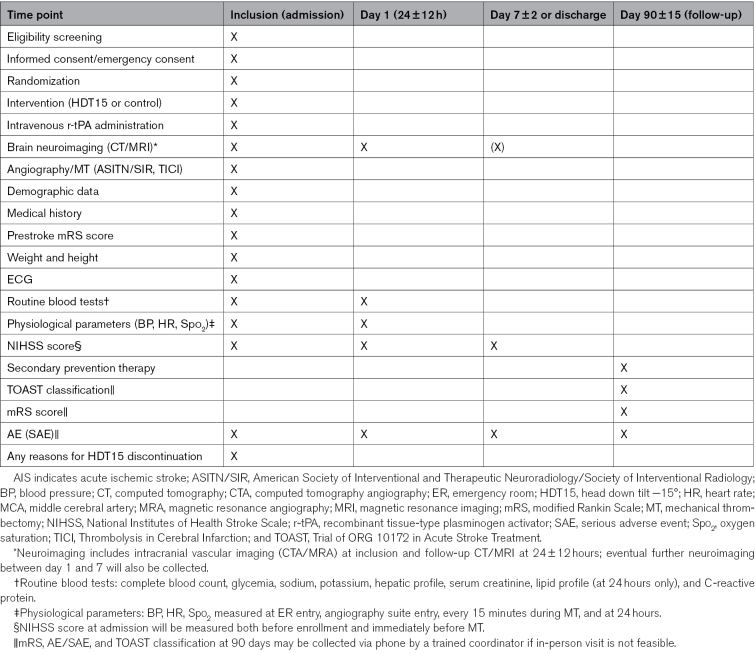
Study Procedures for Patients With a Target Diagnosis of AIS With MCA-M1 Occlusion

### Randomization

Patients with suspected AIS and confirmed isolated MCA-M1 occlusion via computed tomography angiography or magnetic resonance angiography, who are assigned to undergo MT, will be screened for eligibility by the treating neurologist and interventional neuroradiologist.

Eligible patients will be centrally randomized using a secure, web-based software application immediately after obtaining written informed consent (or emergency consent) and admission neuroimaging. Randomization will follow a preestablished list prepared by the study statistician, with a 1:1 allocation ratio between the HDT15 (intervention) and usual positioning (control) groups. Randomization will be stratified by site and baseline National Institutes of Health Stroke Scale (NIHSS) score (dichotomized 0–5 versus ≥6), assessed at presentation in the emergency room (ER) by the trained treating neurologist before treatment allocation. An NIHSS score of 0 to 5, indicating a minor stroke despite M1 occlusion, will be used as a surrogate marker of good baseline collateral status, accounting for interindividual heterogeneity.^22^ Randomization and patient data will be stored securely in compliance with data protection regulations.

### Informed Consent, Emergency and Deferred Consent Procedures

Patients with AIS due to left or right M1 occlusion will be identified at admission after routine neuroimaging. Inclusion and exclusion criteria will be assessed based on information available at admission. The decision to enroll a patient will be based on an appropriate benefit-risk balance. Whenever possible, written informed consent will be obtained from the patient or from a legally authorized representative before inclusion, in accordance with intracranial hemorrhage–GCP guidelines (Good Clinical Practice).

If the patient is unable to provide consent and no representative is available, an emergency consent procedure will be applied in compliance with EU Regulation 536/2014 (section 36) and 2025 AIFA guidelines (The Italian Medicines Agency). In this situation, the patient’s next of kin will be informed whenever possible, while the investigator will document and sign the emergency consent procedure in the medical record. Deferred written consent from the patient or his/her legal representative will then be obtained as soon as the clinical condition allows. Patient assent will be explored in all cases, and refusal will be respected. Further details are reported in the Supplemental Material.

### Intervention

HDT15 will be continuously applied in the intervention group across 2 settings: (1) in the ER, by tilting the stretcher to lower the head by −10° to −15°, verified by using a digital inclinometer or mobile phone app (Figure 1A); (2) in the angiography suite, by similarly tilting the angiography table to −10° to −15°, automatically verified by the angiography system. The precise degree of tilt will vary within this range based on the equipment at each clinical site. Staff at all sites will be trained to ensure consistent application and measurement of HDT15. HDT15 will start immediately after randomization in the ER (after vascular neuroimaging) and will be maintained during transfer to the Angiography Suite and throughout the thrombectomy procedure, until the patient is transferred from the angiography table to their bed in a standard flat position (0°). The intervention will not delay standard care, as the in-hospital pathway—including transfer from the neuroimaging room to the angiography suite and MT procedural steps—provides sufficient time for HDT15 application. The duration of HDT15 is expected to range from 30 to 90 minutes, from initiation to the assessment of the primary efficacy end point. This duration aligns with preclinical evidence suggesting detectable hemodynamic changes within this timeframe.^13,15^ Although we expect high tolerability, HDT15 will be discontinued in cases of patient-reported intolerable discomfort, observed severe discomfort in noncommunicative patients, or at the treating physician’s discretion for clinical reasons (eg, vomiting). All reasons for discontinuation will be recorded and reported as safety data.

Patients in the control group reflect current clinical practice^16^: during the ER phase, they will be maintained in the usual position (HOB ranging from 0° to +30°; Figure 1B and 1C), with positioning at the discretion of the treating physician due to absent guideline recommendations, and during the MT procedure they will lay at 0° on the angiography table. The exact angle will be verified using a digital inclinometer or mobile phone app in the ER phase and automatically by the angiography system during the MT procedure. MT will be performed as per usual care in both groups.

HOB angles and durations will be recorded in both arms to monitor protocol adherence. Although a minority of patients with HDT15 may require elevation to standard positioning (0° to +30°) due to intolerance, crossover from the control arm to HDT15 is unlikely, as this is not standard practice in participating centers. In addition, despite conscious sedation being the preferred approach for MT in participating centers, for patients needing intubation, the HOB will be briefly set to 0° during tube placement to ensure safety, with immediate repositioning to the assigned position (HDT15: −10° to −15°; control: 0° to +30°) postintubation to maintain protocol adherence.

### Study Procedures

After randomization and intervention allocation, the degree of tilt (HDT15: range from −10° to −15°; control: range from 0° to +30°) and any adverse events will be recorded. At admission, demographic data, medical history, site of M1 occlusion, use of intravenous thrombolysis, anesthesia method, blood pressure, and MT procedural details (eg, devices, Thrombolysis in Cerebral Infarction score) will be collected. The inclusion visit will include clinical (vital signs) and neurological assessments (NIHSS score at admission in the ER and immediately before MT, prestroke modified Rankin Scale [mRS]), serious adverse event evaluation, a 12-lead ECG, routine blood tests, and neuroimaging (computed tomography or magnetic resonance imaging with vascular imaging, and digital subtraction angiography during MT). Safety monitoring includes arterial oxygen saturation (Sao_2_) and other vital parameters, with measurements at angiosuite entry and during MT, along with neurological status, monitored from randomization to MT completion, recording of HOB angles and treatment durations in both arms (HDT15: −10° to −15°; control: 0° to +30°). Reasons for HDT15 discontinuation and technical challenges related to MT will be recorded.

At 24 hours, a neurologist will perform a clinical and neurological examination (NIHSS score), assess serious adverse events, and conduct routine blood chemistry tests. Follow-up neuroimaging (computed tomography or magnetic resonance imaging) will monitor stroke evolution and detect early complications, such as symptomatic intracranial hemorrhage. On day 7 or hospital discharge (whichever occurs first), a neurologist will conduct a neurological examination (NIHSS score) and assess serious adverse events. At 90 days, a standardized assessment by a neurologist will evaluate neurological disability (mRS score), stroke cause (TOAST [Trial of ORG 10172 in Acute Stroke Treatment] classification), and serious adverse events. If an in-person visit is not feasible, a phone call by a trained study coordinator will collect the mRS score and serious adverse event data. Data will be recorded in a secure electronic case report form. Study procedures are detailed in Table 2.

### Primary and Secondary Outcomes

The primary end point is the achievement of good collateral status, defined as grade 3 or 4 on the American Society of Interventional and Therapeutic Neuroradiology/Society of Interventional Radiology (ASITN/SIR) collateral scale (ranging from 0, no collaterals, to 4, excellent collaterals), assessed immediately before MT.^23^ This will be evaluated by a blinded central imaging core laboratory using pretreatment diagnostic angiographic runs, performed as the first procedural step of MT. Technical details of the ASITN/SIR scale and assessment procedures are provided in the Supplemental Material.

Patients achieving complete or partial recanalization (spontaneous or r-tPA [recombinant tissue-type plasminogen activator]-induced) at the first angiographic assessment during MT will be excluded from the primary outcome analysis, because the ASITN/SIR score could not be assigned.

Secondary end points will assess feasibility, safety, and efficacy outcomes, evaluated by investigators blinded to treatment allocation. Patients and family members will be instructed not to disclose treatment allocation during visits or interviews. Feasibility outcomes include the proportion of patients maintaining HDT15 until completion of MT, encompassing patient tolerance, the ability to perform MT at −10° to −15° tilt, and any other reasons for premature treatment termination, and hospital admission-to-arterial access time. Safety outcomes include physiological parameters (systolic and diastolic blood pressure, oxygen saturation) measured at ER entry, Angiography Suite entry, and every 15 minutes during MT, proportion of patients experiencing vomiting during treatment, neurological deterioration (≥4-point NIHSS score increase within 24 ± 12 hours), symptomatic intracranial hemorrhage (symptomatic intracranial hemorrhage) per SITS-MOST (Safe Implementation of Thrombolysis in Stroke-Monitoring Study) definition within 24 ± 12 hours, and pneumonia within 72 hours postrandomization. Safety data, including serious and nonserious adverse events, as well as reasons for HDT15 discontinuation (eg, discomfort, vomiting, neurological worsening, headache, respiratory distress) and technical challenges related to MT will be reported for both treatment arms. Efficacy outcomes include functional outcome at 3 months, assessed by the mRS score, and early neurological improvement before MT, at 24 ± 12 hours and at 7 days or discharge (whichever comes first), defined as percent change in NIHSS score ([admission NIHSS score−time point NIHSS score]×100/admission NIHSS score). Details of secondary end points are provided in Table 3.

**Table 3.**
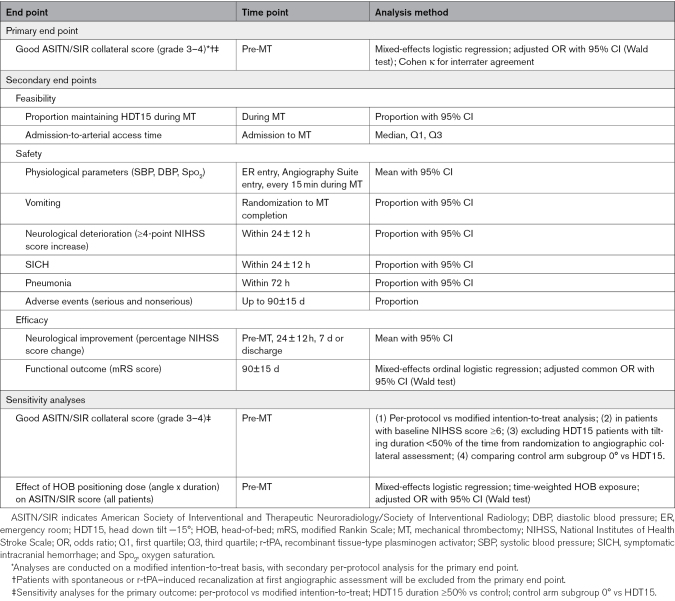
Primary and Secondary End Points

### Data and Safety Monitoring Board

An independent data and safety monitoring board, comprising a neurologist, a biostatistician, and stroke patients association representatives, will oversee participant safety, study conduct, and progress. The data and safety monitoring board will have full access to trial data, meet every 6 months and additionally on-demand, and provide recommendations to the Steering Committee regarding the trial’s continuation, modification, or termination.

### Sample Size Estimation

Based on prior data on the distribution of patients with acute stroke across the ASITN/SIR collateral scale,^24^ a sample size of 118 patients (59 per group) provides 80% power to detect a 25% difference in good collateral status (60% in experimental group versus 35% in control group), considered clinically significant, with a 2-sided alpha of 0.05.

### Statistical Analysis

Analyses will be conducted on a modified intention-to-treat basis (after exclusion of patients achieving complete or partial recanalization at the first angiographic assessment during MT), with a secondary per-protocol analysis of the primary end point. The per-protocol analysis will include only patients who adhered to the assigned intervention without major protocol deviations. Safety and feasibility will be assessed in patients who received the assigned intervention. The primary end point (ASITN/SIR collateral score, grade 3–4) will be analyzed using a mixed-effects logistic regression model, adjusting for baseline NIHSS score (0–5 versus ≥6, fixed effect) and site (as random effects) and, with the effect of intervention group (vs control) quantified by the odds ratio and 95% CI via a Wald test. Baseline collateral status on vascular neuroimaging at admission (Tan score, dichotomized as 0%–50% versus >50%–100% collateral supply filling), and intravenous r-tPA treatment (yes/no) will be included as covariates in the primary end point analysis. This approach will be used for the following secondary analyses on the primary end point. A preplanned secondary analysis will evaluate the effect of HOB positioning on the primary end point (ASITN/SIR collateral score, grade 3–4) across all patients, treating HOB angle as a continuous variable calculated as a time-weighted average (integrating angle and duration per patient to account for within-patient variations, including HDT15 patients requiring standard positioning due to intolerance). Pre-specified sensitivity analyses will include: (1) per-protocol versus modified intention-to-treat analysis; (2) inclusion of only those patients with baseline NIHSS score ≥6; (3) exclusion of HDT15 patients with tilting duration <50% of the time from randomization to angiographic collateral assessment; and (4) comparison of control arm subgroup 0° versus HDT15.

The number of patients achieving complete or partial recanalization (spontaneous or r-tPA-induced) at the first angiographic assessment during MT will be reported for both arms.

For secondary end points, feasibility and safety proportions will be reported with 95% CIs, continuous safety variables (eg, arterial blood pressure) as means with 95% CI, and time-to-arterial puncture as median with interquartile range. The mRS at 3 months will be analyzed using a mixed-effects ordinal logistic regression model, adjusting for intervention group, site (random effect) and baseline NIHSS score (fixed effect), with the effect quantified by the adjusted common odds ratio and 95% CI. An interim analysis of the primary and secondary outcomes is planned 12 months after the start of recruitment. Based on the results of this analysis and the progress of enrollment, the Steering Committee may consider a reassessment of the sample size and a recalculation of the study’s statistical power. Missing data will not be imputed. Analyses will be performed using R and SAS software by the biostatistics unit of the University of Milano-Bicocca. Further details are provided in the Supplemental Material.

## Discussion

In the DOWN-SUITE trial, we investigate HDT15 as a novel collateral therapeutic to acutely increase cerebral blood flow in patients with LVO-AIS eligible for MT. By enhancing penumbra survival, HDT15 aims to extend the therapeutic time window and improve the efficacy of recanalization therapies, ultimately leading to better clinical outcomes. The DOWN-SUITE trial’s focus on the ASITN/SIR collateral score (grade 3–4) as the primary end point addresses a critical mechanistic gap in understanding HDT15’s effect on leptomeningeal collateral flow in MCA-M1 occlusion patients. As a proof-of-concept study, DOWN-SUITE is a critical step before moving to larger clinical trials, enrolling broader patient populations. The control arm (any range between 0° and +30°) aligns with current clinical practice variability. Notably, HOB angles will be captured, and a gradual shift toward 0° is expected in the control arm, after the results of the ZODIAC trial, enhancing the study’s relevance to evolving standards.^18^ Importantly, we will perform a dedicated analysis treating the time-weighted average head-position angle as a continuous variable, which may allow identification of a threshold at which changes in angle begin to enhance collateral flow. This proof-of-concept randomized controlled trial primarily seeks to first demonstrate HDT15’s superiority in collateral recruitment before MT compared with standard HOB positioning (0° to +30°) by direct catheter angiography.

Given that the MCA territory receives collateral blood supply from both the anterior and the posterior cerebral arteries, and anterograde blood flow from the ipsilateral internal carotid artery is required for a proper visualization of collaterals during transcatheter angiography, we selected isolated MCA-M1 occlusions (excluding [1] concomitant internal carotid artery occlusion at any level and [2] patients with ipsilateral A1 segment hypoplasia) to maximize the potential for leptomeningeal collateral recruitment. Secondary objectives include providing robust evidence on the safety, feasibility, and clinical efficacy of HDT15. The DOWN-SUITE trial’s novel evaluation of HDT15 addresses a critical gap in knowledge in lower head positioning as a collateral flow modifier in MCA-M1 occlusion patients undergoing MT. Unlike HeadPoST and ZODIAC,^17,18^ which established the safety and efficacy of HOB 0° versus +30° positioning, HDT15 may accelerate collateral recruitment compared with HOB 0°, potentially enhancing penumbral survival in MT-eligible patients. However, HDT15’s greater impact on venous outflow and intracranial pressure limits the extrapolation of safety data from these 0°/+30° studies. Similarly, the HOPES2 trial (Head-Down Position for Acute Ischemic Stroke With Large Artery Atherosclerosis),^20^ which suggested safety and efficacy of head down positioning in patients ineligible for recanalization (eg, with hemodynamic stroke due to proximal or intracranial stenosis), differs from DOWN-SUITE’s MT-eligible cohort. These distinctions highlight DOWN-SUITE’s unique contribution to optimizing collateral flow in AIS management.

The DOWN-SUITE trial has strengths and limitations. The randomized, multicenter design with blinded imaging and clinical evaluations, and the use of the validated ASITN/SIR collateral scale as the primary end point, will guarantee reliable data and consistency of collateral evaluation. The in-hospital setting of HDT15 application will support patients’ safety in all phases of the trial. However, the open-label design may potentially introduce bias in the initial assessment phase, although this risk is mitigated by standardized in-hospital care pathways for AIS. The focus on MCA-M1 occlusions, while optimizing collateral recruitment potential, limits generalizability to other LVO causes. In addition, the relatively short duration of HDT15 application (estimated between 30 and 90 minutes) may be insufficient to capture subtle or delayed changes in collateral recruitment, given the unknown speed of collateral recruitment in humans. Finally, the sample size of 118 patients might be underpowered to detect differences in secondary end points, such as functional outcomes at 90 days, or to fully account for confounding factors like onset-to-treatment time variability.

If HDT15 proves effective in enhancing collateral circulation, DOWN-SUITE will lay the foundation for larger randomized controlled trials in a broader stroke population, including patients transferred from spoke hospitals to hub stroke centers for thrombectomy and unselected patients with suspected AIS in the prehospital setting. The ultimate goal is to establish HDT15 as a practice-changing, standard-of-care emergency treatment for hyperacute stroke, easily implementable within international AIS management guidelines and stroke care systems globally.

## Conclusions

The DOWN-SUITE trial is a phase 2a/b, proof-of-concept, multicenter, prospective, randomized, open-label clinical trial with blinded outcome assessment, designed to investigate the effect of HDT15 in enhancing collateral circulation in patients with LVO-AIS before MT. By targeting MCA-M1 occlusions, the trial aims to demonstrate HDT15’s superiority in collateral recruitment compared with standard positioning (0° to +30°), while secondarily assessing its safety, feasibility, and clinical efficacy. As the first study to evaluate HDT15 against usual head positioning in patients with LVO-AIS undergoing MT, DOWN-SUITE addresses a critical unmet need in acute stroke care. Given its simplicity, low cost, and ease of implementation, HDT15 has the potential to become an evidence-based collateral therapeutic for AIS, paving the way for larger trials and possible integration into international stroke management guidelines to improve patient outcomes globally.

## ARTICLE INFORMATION

### Acknowledgments

Drs Beretta and Ferrarese contributed to this work during their personal involvement in the Italian Ministry of University and Research Dipartimenti di Eccellenza 2023-2027.

### Sources of Funding

This project was supported by Ministero dell’Università e della Ricerca (NextGenerationEU, grant 2022LNL3H3, PNRR M4.C2.I1.1, avviso 104/2022, and CUP H53D23005490006), which has no role in study design, conduct, data analysis, or reporting.

### Disclosures

None.

### Supplemental Material

Supplemental Methods

Full-Length Study Protocol

SPIRIT Checklist

References 23,^25^

## Supplementary Material


