# Supplementary material for: Head Down Tilt 15° to Increase Collateral Flow in Acute Ischemic Stroke: Rationale and Study Protocol of a Multicenter, Randomized, Proof-of-Concept, Phase 2a/b Trial in Patients Treated With Mechanical Thrombectomy (DOWN-SUITE)
Source: Stroke Vasc Interv Neurol. 2026 Feb 4;6(2):e002221. doi: 10.1161/SVIN.125.002221 (PMC12959440; doi:10.1161/SVIN.125.002221)
Supplement: Supplementary file 1 [file svi2-6-e002221-s001.pdf]

SUPPLEMENTAL MATERIAL

Title: Head down tilt 15° to increase collateral flow in acute ischemic stroke: rationale and study protocol of a multicenter, randomized, proof of concept, phase 2a/b trial in patients treated with mechanical thrombectomy (DOWN-SUITE)

Francesco Andrea Pedrazzini, MD[1], Lorenzo Piergallini, MD[2], Susanna Diamanti, MD, PhD[1,2], Enrico Fainardi, MD, PhD[3], Sergio Lucio Vinci, MD[4], Caterina Sozzi, MD[5], Matteo Faré, MD[5], Emanuela Rossi, PhD[5], Francesca Graziano, PhD[6], Francesca Poggetti, MD[5], Gabriele Mainini, MD[5], Angela Giglio, MD[5], Andrea Magi, MD[5], Giulia Pederzoli, MD[5], Agnese Anzani, MD[5], Elisabetta De Bernardi, PhD[5], Valeria Cerina, PhD[5], Tae-Hee Cho, MD, PhD[7], Fabien Chauveau, PhD[8], Davide Carone, MD, PhD[9], Gianpaolo Basso, MD, PhD[5], Giuseppe Citerio, MD[2,5], Cristina Sarti, MD, PhD[10], Nicola Limbucci, MD[11], Francesco Janes, MD, PhD[12], Carmela Casella, MD[13], Antonio Toscano, MD[13], Simona Sacco, MD[14], Danilo Toni, MD[15], Paolo Remida, MD[2], Carlo Ferrarese, MD, PhD[1,2], Simone Beretta, MD, PhD[1,2]

[1]Department of Medicine and Surgery and Milan Center for Neuroscience (NeuroMI), University of Milano-Bicocca, Monza, Italy

[2]Department of Neuroscience, Fondazione IRCCS San Gerardo dei Tintori, Monza, Italy

[3]Neuroradiology Unit, Department of Experimental and Clinical Biomedical Sciences, University of Florence, Careggi University Hospital, Florence, Italy

[4]Neuroradiology Unit, University of Messina, Italy

[5]Department of Medicine and Surgery, University of Milano-Bicocca, Monza, Italy

[6]Biostatistics and Clinical Epidemiology, Fondazione IRCCS San Gerardo dei Tintori, Monza, Italy

[7]Department of Vascular Neurology, Hospices Civils de Lyon, Lyon, France

[8]Lyon Neuroscience Research Center, CNRS, INSERM, Univ. Lyon 1, Lyon, France

[9]Acute Vascular Imaging Centre, Radcliffe Department of Medicine, University of Oxford, Oxford, United Kingdom

[10]NEUROFARBA Department, Neuroscience Section, University of Florence

[11]Neuroradiology Unit, Azienda Ospedaliero-Universitaria Careggi, Firenze, Italy

[12] Department of Medicine, University of Udine and Clinical Neurology, Udine University Hospital, Italy

[13]UOSD Stroke Unit, AOU G. Martino, Messina Italy

[14]Department of Biotechnological and Applied Clinical Sciences, University of L'Aquila, L'Aquila, Italy

[15]Department of Human Neurosciences, University of Rome La Sapienza, Rome, Italy

Supplemental methods

A. Primary endpoint – Technical aspects 3
B. Statistical methods 4
   General considerations 4
   Primary endpoint 5
   Secondary endpoints 6
C. Informed consent, emergency and deferred consent procedures 8

A. Primary endpoint – Technical aspects

The primary endpoint is the achievement of a good collateral status, defined as grade 3 or 4 on the ASITN/SIR scale. This scale is the widely accepted reference method for grading the collateral circulation in AIS patients, using the gold-standard method of digital subtraction angiography. The standardized technical aspects were detailed in the position paper of the ASITN/SIR[23], and are reproduced here:

Table 1. Angiographic collateral flow grading system.

Grade	Angiographic findings
Grade 0	No collaterals visible to the ischemic site
Grade 1	Slow collaterals to the periphery of the ischemic site with persistence of some of the defect
Grade 2	Rapid collaterals to the periphery of ischemic site with persistence of some of the defect and to only a portion of the ischemic territory
Grade 3	Collaterals with slow but complete angiographic blood flow of the ischemic bed by the late venous phase
Grade 4	Complete and rapid collateral blood flow to the vascular bed in the entire ischemic territory by retrograde perfusion

In order to strictly follow the ASITN/SIR grading recommendations detailed above, the acquisition parameters of the angiography runs for assessing the primary endpoint will be specified and homogenized across all recruiting centers (anterior and lateral field of view, frame rate, inclusion of arterial and late venous phases).

The Central Imaging Core Lab will also evaluate the baseline neuroimaging and determine the pretreatment collateral status of all patients, using a dichotomized version of the criteria reported by Tan et al.[25] (collateral supply filling 0% to 50% versus 50% to 100% of the occluded MCA territory).

Two independent experts (Interventional Neuroradiologists), blind to treatment allocation and not involved in patient recruitment will independently grade all patients, thus allowing for the evaluation of inter-rater reproducibility. All discrepant cases will be resolved by a third independent expert, also blind to treatment allocation.

B. Statistical methods

General considerations

The quantitative variables will be described by the following parameters: number of patients, number of missing values, mean, standard deviation (SD), median, first and third quartiles (Q1 and Q3), minimum and maximum. Categories could be defined if applicable using a cut-off threshold from literature or quantiles.

The qualitative variables will be described by the following parameters: number of patients, number of missing values, frequency and percentage of each modality (missing values will not be included in the denominator used for frequency computation).

The analyses will be performed using the R and SAS software. They will be performed by the biostatistics unit of the University of Milano-Bicocca.

Primary endpoint

The analysis of the primary endpoint will be performed by a mixed effects logistic regression model. It will take into account as explanatory variables the group of intervention, as well as the stratification factors (the site as random effects and the baseline NIHSS score [dichotomized 0 to 5 versus 6 or more] as fixed effect) and will include as covariates baseline collateral status (Tan score, dichotomized as 0-50% versus >50%-100% collateral supply filling) and intravenous rt-PA treatment (yes/no). The effect of the intervention on the occurrence of good collaterals will be assessed by a Wald test and quantified through the adjusted odds ratio with the associated 95% confidence interval.

The agreement between readers will be estimated using the Cohen's kappa or the weighted Cohen's kappa coefficient for the dichotomized ASITN/SIR collateral score (good collaterals: score 3 to 4; poor collaterals: score 0 to 2) and the ASITN/SIR collateral ordinal score, respectively. Coefficients of ≤0.20, 0.21-0.40, 0.41-0.60, 0.61-0.80 and >0.80 indicate poor, fair, moderate, good and excellent agreement, respectively.

Pre-specified sensitivity analyses for the primary endpoint (ASITN/SIR collateral score, grade 3–4) will include: (1) per-protocol vs. modified intention-to-treat analysis; (2) primary outcome in patients with baseline NIHSS ≥6, using a mixed-effects logistic regression model adjusted for stratification factors (site), to evaluate potential greater benefit in those with higher stroke severity; (3) exclusion of HDT15 patients with tilting duration <50% of the time from randomization to angiographic collateral assessment; and (4) comparison of control arm subgroup 0° vs HDT15. Results will report adjusted odds ratios with 95% confidence intervals.

A pre-planned secondary analysis will evaluate the effect of head of bed (HOB) positioning on the primary endpoint (ASITN/SIR collateral score, grade 3–4) across all patients, treating HOB angle as a continuous variable calculated as a time-weighted average (integrating angle and

duration per patient to account for within-patient variations, including HDT15 patients requiring standard positioning due to intolerance). In particular, for each patient, the time-weighted average lower HOB exposure will be calculated as follows, with higher values representing greater exposure to lower head positions:

$$\textit{time-weighted lower HOB exposure} = \left(\sum \frac{(30° - HOB\ angle + 1) \times duration}{total\ duration}\right)$$

This analysis will use a mixed-effects logistic regression model, adjusted for stratification factors (site as random effect, baseline NIHSS [0–5 vs. ≥6] as fixed effect) and covariates (baseline collateral status on vascular neuroimaging at admission; intravenous rt-PA treatment), reporting adjusted odds ratios with 95% confidence intervals.

Secondary endpoints

i. Feasibility outcomes:

   a. The proportion of patients randomized to the intervention group who are able to maintain HDT15 during the entire MT procedure will be calculated with their 95% confidence interval.

   b. The hospital admission-to-arterial access time (i.e. time to arterial puncture) will be described by median, first and third quartiles (Q1 and Q3) for each group.

ii. Safety outcomes:

   c. Mean values of systolic blood pressure, diastolic blood pressure and oxygen saturation at entry in the Emergency Room, at entry in the angio suite and every 15 minutes from the start to the end of MT; they will be treated as continuous variables and calculated as mean with their 95% confidence interval for each group.

d. Proportion of patients presenting one or more episodes of vomiting from randomization to the completion of MT will be calculated with their 95% confidence interval for each group.

e. Reasons for HDT15 discontinuation (e.g., intolerable discomfort, observed severe discomfort in non-communicative patients, vomiting, neurological worsening, headache, or respiratory distress), minor adverse events, and MT-related technical challenges will be recorded and reported as proportions with 95% confidence intervals.

f. The proportions of patients who had an increase of ≥ 4 points on the NIHSS score within 24±12 hours of the randomization will be calculated with their 95% confidence interval for each group.

g. The proportions of patients who had SICH within 24±12 hours will be calculated with their 95% confidence interval for each group.

h. The proportions of patients with pneumonia within the first 72 hours after randomization will be calculated with their 95% confidence interval for each group.

i. Safety data, including serious and non-serious adverse events, as well as reasons for HDT15 discontinuation (e.g. discomfort, vomiting, neurological worsening, headache, respiratory distress) and technical challenges related to MT will be reported for both treatment arms. Adverse events will be categorized according to the Medical Dictionary for Regulatory Activities (MedDRA) v28.0.

iii. Efficacy outcomes:

- Percent change in NIHSS ([(Admission NIHSS – time-point NIHSS) × 100 / Admission NIHSS]) from admission to immediately pre-MT, at 24±12 hours, and at 7±2 days (or discharge), calculated with 95% confidence intervals.

- Functional outcome at 3 months, assessed with the ordinal score on the modified Rankin scale (shift across outcomes on the mRS between groups).

The analysis of the effect of the group on the mRS at 3 months will be performed by the mixed effects ordinal logistic regression model. It will take into account as explanatory variables the group of intervention, as well as the stratification factors (the site and the baseline NIHSS score [dichotomized 0 to 5 versus 6 or more] as random effects). The effect of the intervention on the occurrence of good collaterals will be assessed by a Wald test and quantified through the adjusted common odds ratio with the associated 95% confidence interval.

C. Informed consent, emergency and deferred consent procedures

Patients eligible for enrolment will be identified at admission, once routine neuroimaging has been completed. All patients with AIS due to left or right M1 occlusion will be screened for participation. Inclusion and exclusion criteria will be assessed based on information available at admission.

The investigator’s decision to enroll a patient must be based on an appropriate benefit–risk balance. The patient, or his/her legally authorized representative, will be fully and fairly informed, in understandable terms, about the objectives and constraints of the study, the possible risks, the required monitoring and safety measures, and the right to refuse participation or to withdraw at any time. This information is provided in an information and consent form, and written informed consent will be obtained by the investigator prior to inclusion.

If the patient is unable to provide informed consent and no legally authorized representative is available at the time of enrollment, an emergency consent procedure (EU 536/2014, section 36; 2025 AIFA guidelines) will be applied. Considering the AIS emergency situation, the time-dependent nature of the experimental treatment, and the favorable safety profile of HDT15, the investigator will inform the patient's next of kin, if available, about the study. In such cases, the investigator documents in the medical record that the emergency consent procedure has been applied and signs the relevant documentation. The patient or his/her legal representative must then provide full written informed consent as soon as the patient's clinical condition allows it (deferred consent). Emergency consent must always be documented before performing any clinical or paraclinical examination required by the study. In all cases, the patient's assent will be explored. If the investigator has reason to believe that the patient would refuse the procedure, the treatment will not be administered.

If the patient, or the legally authorized representative (or the next of kin in case of emergency consent), decides to withdraw from the trial, the patient will be discontinued from the study. In all cases, the investigator must record the progress of the inclusion in the medical file, and written informed consent must be obtained as soon as the patient's clinical condition allows it.
