# Supplementary material for: Head Down Tilt 15° to Increase Collateral Flow in Acute Ischemic Stroke: Rationale and Study Protocol of a Multicenter, Randomized, Proof-of-Concept, Phase 2a/b Trial in Patients Treated With Mechanical Thrombectomy (DOWN-SUITE)
Source: Stroke Vasc Interv Neurol. 2026 Feb 4;6(2):e002221. doi: 10.1161/SVIN.125.002221 (PMC12959440; doi:10.1161/SVIN.125.002221)
Supplement: Supplementary file 3 [file svi2-6-e002221-s003.pdf]

Head down tilt 15° to increase collateral flow in acute ischemic stroke: a multicenter, randomised, proof of concept, phase 2a/b trial in patients treated with mechanical thrombectomy

DOWN-SUITE

Version 5 of 04/10/2025

Sponsor:

University of Milano-Bicocca
Department of Medicine and Surgery
Via Cadore 48, 20900 Monza (MB)
ITALY

Chief Investigator:

Dr. Simone Beretta, MD, PhD
Department of Medicine and Surgery
University of Milano-Bicocca
Via Cadore 48, 20900 Monza (MB)
ITALY
Phone: +39 02 6448 8128
E-mail: simone.beretta@unimib.it

SUMMARY

Title	Head down tilt 15° to increase collateral flow in acute ischemic stroke: a multicenter, randomised, proof of concept, phase 2a/b trial in patients treated with mechanical thrombectomy
Acronym	DOWN-SUITE
Protocol version	Version 5.0
Protocol date	04/10/2025
Type of study	Clinical study of non-medicinal product
Document status	Final
Sponsor	University of Milano-Bicocca<br>Department of Medicine and Surgery<br>Via Cadore 48, 20900 Monza (MB)<br>ITALYITALY

Confidentiality statement:
This document contains confidential information belonging to the University of Milano-Bicocca. Except as may be otherwise agreed to in writing, by accepting or reviewing these materials, you agree to hold such information in confidence and not to disclose it to others (except where required by applicable law), nor to use it for unauthorised purposes. In the event of actual or suspected breach of this obligation, Milano Bicocca University should be promptly notified.

PROTOCOL SIGNATURE PAGE:

**Chief Investigator of the study**	
Name and Surname: Simone Beretta<br><br>Academic title: Assistant Professor of Neurology<br><br>Department: Medicine and Surgery<br><br>Institute: University of Milano-Bicocca<br><br>City, Country: Monza, Italy	
Signature<br>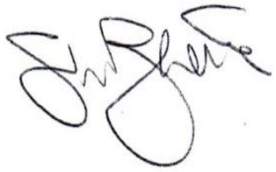	Date 04/10/2025

**Principal investigator at clinical site**	
Name and Surname:<br><br>Academic title:<br><br>Department:<br><br>Institute:<br><br>City, Country:	
Signature	Date

LIST OF ABBREVIATIONS

**AE**	Adverse Event
**AIS**	Acute Ischemic Stroke
**CT**	Computerised tomography
**CRF**	Case Report Form
**DSMB**	Data Safety Monitoring Board
**ECG**	Electrocardiography
**GCP**	Good Clinical Practice
**GDPR**	General Data Protection Regulation
**HDT15**	Head Down Tilt 15°
**ICH**	International Conference on Harmonization
**ITT**	Intention-to-treat
**IVT**	Intravenous Thrombolysis
**IWRS**	Interactive Web Response System
**LVO**	Large Vessel Occlusion
**MCA**	Middle Cerebral Artery
**MRI**	Magnetic Resonance Imaging
**mRS**	modified Rankin Scale
**MT**	Mechanical Thrombectomy
**NIHSS**	National Institutes of Health Stroke Scale
**SAE**	Serious Adverse Event
**SD**	Standard Deviation
**SICH**	Symptomatic Intracranial Hemorrhage
**TOAST**	Trial of ORG 10172 in Acute Stroke Treatment

TABLE OF CONTENTS

1. GENERAL INFORMATION 8

1.1 FLOW CHART 8
1.2 PROJECT IDENTIFIERS 9
1.3 SPONSOR 9
1.4 INVESTIGATORS 9
1.4.1 Coordinating investigator 9
1.4.2 Associate investigators 9
1.5 ASSOCIATE SCIENTISTS 10
1.6 METHODOLOGIST – BIOSTATISTICIAN 10
1.7 DATA MANAGEMENT 10
1.8 COORDINATION CENTER 10
1.9 COMMITTEES 11
1.9.1 Steering Committee 11
1.9.2 Data and Safety Monitoring Board 12
1.9.3 Central Imaging Core Lab 12

2 SCIENTIFIC JUSTIFICATION 13

2.1 THE CURRENT STATE OF KNOWLEDGE - RATIONALE 13
2.2 RESEARCH HYPOTHESIS 14
2.3 JUSTIFICATION OF THE METHODOLOGICAL CHOICES 14
2.4 TARGET POPULATION 14
2.5 BENEFIT/RISK RATIO 14
2.6 EXPECTED BENEFITS 15

3 OBJECTIVES OF THE RESEARCH 16

3.1 MAIN OBJECTIVE 16
3.2 SECONDARY OBJECTIVE 16

4 RESEARCH DESIGN 16

4.1 TYPE OF STUDY 16
4.2 METHOD FOR RANDOMISATION 16
4.3 ENDPOINTS 17
4.3.1 Primary endpoint 17
4.3.2 Secondary endpoints 17

5 ELIGIBILITY CRITERIA 18

5.1 INCLUSION CRITERIA 18
5.2 EXCLUSION CRITERIA 18
5.3 PREMATURE STUDY EXIT CRITERIA 18
5.4 CRITERIA FOR DISCONTINUATION OF STUDY PROCEDURE 18
5.5 RECRUITMENT PROCEDURES AND FEASIBILITY 19

6 EXPERIMENTAL STRATEGIES 20

6.1 STUDY STRATEGY: EXPERIMENTAL GROUP 20
6.1.1 Description of the strategy 20
6.1.2 Justification of the chosen strategy 20
6.1.3 Description of contraindications 20
6.2 STRATEGY OF COMPARISON : CONTROL GROUP 21

6.2.1 Description of the strategy ... 21
6.2.2 Justification of the chosen strategy ... 21
6.2.3 Description of contraindications ... 21
6.3 Blinding ... 21
6.3.1 Organisation ... 21
6.3.2 Unblinding ... 21
6.4 Permitted and prohibited associated treatments ... 21

7 GENERAL ORGANISATION ... 22

7.1 Schedule of the study ... 22
7.2 Summary table ... 23
7.3 Conduct of the study ... 24
7.3.1 Screening - Information and informed consent ... 24
7.3.2 Inclusion visit / Randomization ... 24
7.3.3 Follow-up visits ... 25
7.3.4 Imaging tests required for the project ... 25
7.4 Temporary or permanent cessation rules ... 26

8 SAFETY ASSESSMENT ... 27

8.1 Definitions ... 27
8.1.1 Adverse event (AE) ... 27
8.1.2 Serious adverse event (SAE) or reaction ... 27
8.1.3 Adverse reaction ... 27
8.1.4 Unexpected adverse reaction ... 27
8.1.5 New issue ... 27
8.2 Investigator's responsibilities ... 28
8.2.1 Procedures for detecting and collecting adverse events ... 28
8.2.2 Immediate notification of Serious Adverse Events (SAEs) to the sponsor ... 28
8.2.3 Assessment of the causality of AEs ... 29
8.2.4 Restriction of AEs/SAEs and reporting period of AEs/SAEs ... 29
8.3 Responsibilities of the sponsor ... 30
8.3.1 Declaration to the competent authorities ... 30
8.4 Data Safety Monitoring Board ... 30

9 STATISTICAL ASPECTS ... 30

9.1 Number of subjects needed ... 30
9.2 Analysis population ... 30
9.3 Statistical methods ... 31
9.3.1 General considerations ... 31
9.3.2 Primary endpoint ... 31
9.3.3 Secondary endpoints ... 32
9.4 Intermediate analyses ... 33
9.5 Method of taking missing data into account ... 33
9.6 Managing changes to the analysis plan ... 33

10 CENTRAL IMAGING CORE LAB ... 35

10.1 Role ... 35
10.2 Technical aspects ... 35

11 ACCESS RIGHTS TO SOURCE DATA AND DOCUMENTS ... 36

11.1 Access to data ... 36
11.2 Data collected ... 36
11.3 Source documents ... 36
11.4 Data privacy ... 37

12 STUDY MONITORING, QUALITY CONTROL AND ASSURANCE ... 38

13 ETHICAL CONSIDERATIONS ... 38

13.1 COMPETENT AUTHORITIES 38
13.2 SUBSTANTIAL CHANGES 38
13.3 PATIENT INFORMATION AND WRITTEN CONSENT FORM 38
13.4 DECLARATION OF CONFORMITY 39
13.5 EXCLUSION PERIOD 39
13.6 COMPENSATION FOR SUBJECTS AND/OR REGISTRATION IN THE NATIONAL FILE OF PERSONS UNDERGOING INTERVENTION RESEARCH ON HUMAN SUBJECTS OF 1°. 39

14 DATA MANAGEMENT AND RETENTION 40

14.1 CASE REPORT 40
14.2 DATA MANAGEMENT 40
14.3 ARCHIVING 40

15 FUNDING AND INSURANCE 41

15.1 STUDY FUNDING 41
15.2 INSURANCE 41

16 RULES FOR PUBLICATION 41

17 REFERENCES 42

1. GENERAL INFORMATION

1.1 Flow chart

DOWN-SUITE trial flow chart

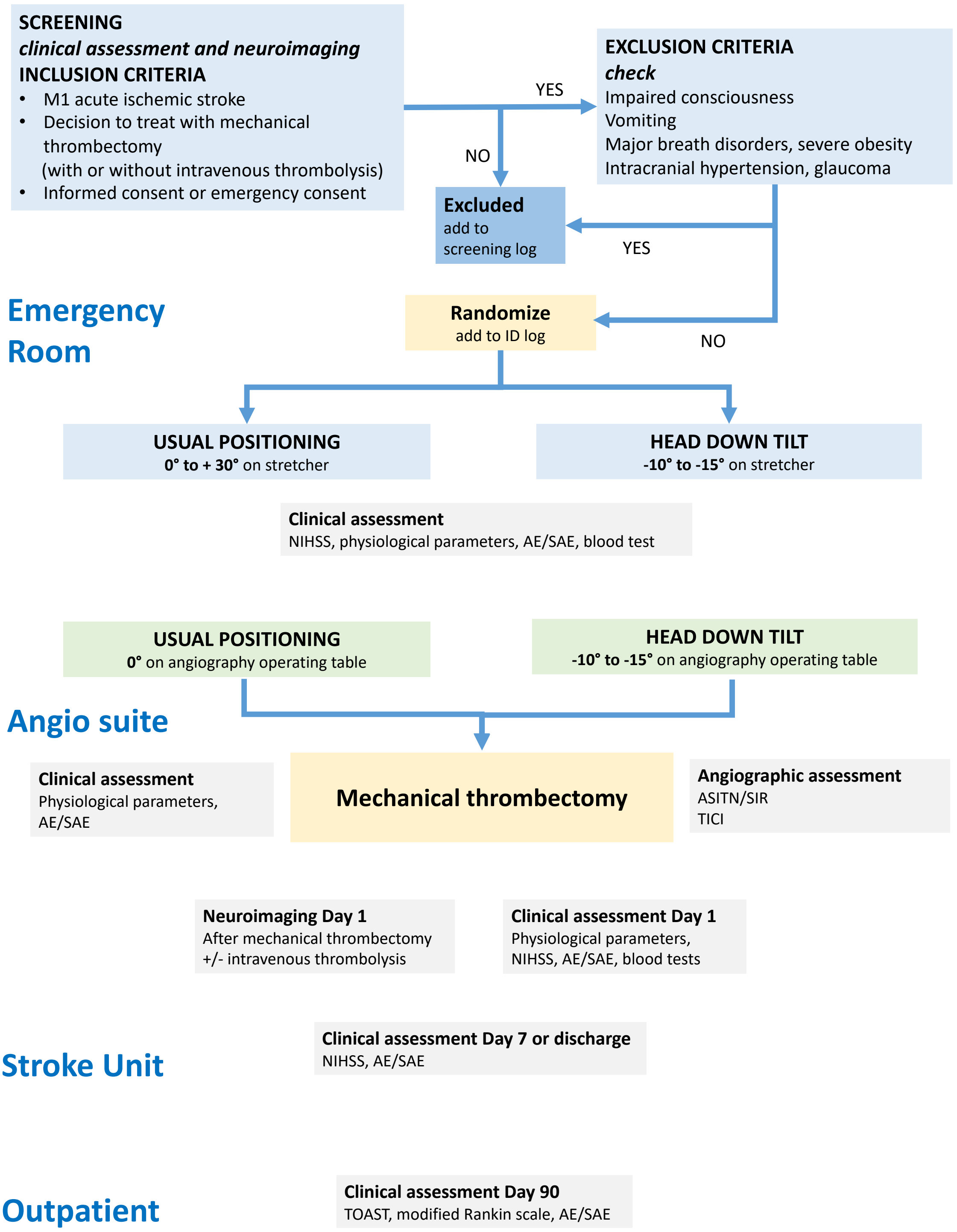

1.2 Project identifiers

Study title: Head down tilt 15° to increase collateral flow in acute ischemic stroke: a multicenter, randomised, proof of concept, phase 2a/b trial in patients treated with mechanical thrombectomy (DOWN-SUITE)
Study ID: DOWN-SUITE
Clinicaltrials.gov registration number: NCT06297863

Version record		
**Version**	**Date**	**Reason for the update**
1	20/05/2024	Initial version
2	02/08/2024	First amendment
3	12/11/2024	Second amendment
4	18/08/2025	Third amendment
5	XX/XX/XXXX	Fourth amendment

1.3 Sponsor

University of Milano-Bicocca
Department of Medicine and Surgery
Via Cadore 48
20900 Monza
ITALY

1.4 Investigators

1.4.1 Chief investigators

Dr. Simone Beretta (Chief Investigator)
Department of Medicine and Surgery
University of Milano-Bicocca
Via Cadore 48
20900 Monza, ITALY
E-mail: simone.beretta@unimib.it

1.4.2 Associate investigators

Prof. Giuseppe Citerio (Neurointensive Care Unit)
Dr. Paolo Remida (Neuroradiology)
Dr. Lorenzo Piergallini (Interventional Neuroradiology)
Department of Neuroscience
Fondazione IRCCS San Gerardo dei Tintori
Via Pergolesi 33
20900 Monza, ITALY

Further associate investigators are listed in the form "Participating Centers".

1.5 Associate scientists

Prof. Tae-Hee Cho
Department of Vascular Neurology
Pierre Wertheimer hospital, Groupement Hospitalier Est, Hospices Civils de Lyon
59 Boulevard Pinel, 69677 BRON Cedex, FRANCE
Email: tae-hee.cho@chu-lyon.fr

Dr. Davide Carone
Acute Vascular Imaging Centre, Radcliffe Department of Medicine, University of Oxford
Headley Way, Headington, Oxford OX3 9DU, UNITED KINGDOM
E-mail: davide.carone@rdm.ox.ac.uk

Dr. Fabien Chauveau
Centre de Recherche en Neurosciences de Lyon (CRNL), CNRS UMR 5292, INSERM U1028, Université Lyon 1, CERMEP – Imagerie du Vivant, Groupement Hospitalier Est
59 Boulevard Pinel, 69677 BRON Cedex, FRANCE
Email: chauveau@cermep.fr

1.6 Biostatistician

- Biostatistician:

Dr. Francesca Graziano
Fondazione IRCCS San Gerardo dei Tintori
Via Pergolesi 33, 20900 Monza - ITALY
E-mail: francesca.graziano@unimib.it

1.7 Data management

BiCRO
Department of Medicine and Surgery, University of Milano-Bicocca
Via Cadore 48, 20900 Monza, ITALY
E-mail: emanuela.rossi@unimib.it

1.8 Coordination Center

Department of Neurology and Stroke Unit
Fondazione IRCCS San Gerardo dei Tintori
Via Pergolesi 33, 20900 Monza, ITALY
E-mail: simone.beretta@unimib.it

1.9 Committees

1.9.1 Steering Committee

The Steering Committee is responsible for all management decisions needed to keep the project focused on its objectives. The Steering Committee will be composed of:

Prof. Carlo Ferrarese (chair)	Neurology and Stroke Unit, Fondazione IRCCS San Gerardo dei Tintori; University of Milano-Bicocca, Monza, Italy
Dr. Simone Beretta (coordinating investigator)	Neurology and Stroke Unit, Fondazione IRCCS San Gerardo dei Tintori; University of Milano-Bicocca, Monza, Italy
Prof. Giuseppe Citerio	Neurointensive Care Unit, Fondazione IRCCS San Gerardo dei Tintori; University of Milano-Bicocca, Monza, Italy
Dr. Paolo Remida	Neuroradiology, Fondazione IRCCS San Gerardo dei Tintori, Monza, Italy
Prof. Enrico Fainardi	Neuroradiology, Careggi Hospital, University of Firenze, Firenze, Italy
Prof. Antonio Toscano	Neurology and Stroke Unit, Policlinico G. Martino, University of Messina, Messina, Italy
Prof. Tae-Hee Cho (independent member)	Vascular Neurology, Hôpital Pierre Wertheimer, Hospices Civils de Lyon, Lyon, France
Dr. Davide Carone (independent member)	Acute Vascular Imaging Centre, Radcliffe Department of Medicine, University of Oxford, Oxford, United Kingdom
Dr. Fabien Chauveau (independent member)	Centre de Recherche en Neurosciences de Lyon, Université Lyon 1, Lyon, France
Miss Nathalie Coingt (independent member)	Patient representative from France AVC (Fédération Nationale France accident vasculaire cérébral)

1.9.2 Data and Safety Monitoring Board

The Data and Safety Monitoring Board will provide expertise by periodic review and evaluation of the accumulated study data for participant safety, study conduct and progress; it will also provide recommendations to the Steering Committee concerning the trial's continuation, modification, or termination.

Dott. Anna Bersano<br>(independent chair)	Cerebrovascular Diseases Unit, Fondazione IRCCS Istituto Neurologico Carlo Besta, Milano, Italy
Dr. Elisa Bianchi<br>(independent member)	Biostatistician, Istituto di Ricerche Farmacologiche Mario Negri, Milano, Italy
Mrs. Emanuela Ceriani<br>(independent member)	Patient representative from ALICe Monza<br>(Associazione per la lotta all'ictus cerebrale)
Mrs. Maria Elena Della Santa<br>(independent member)	Patient representative from ALICe Firenze<br>(Associazione per la lotta all'ictus cerebrale)
Mr. Gianmarco Cannistraci<br>(independent member)	Patient representative from ALICe Messina<br>(Associazione per la lotta all'ictus cerebrale)

1.9.3 Central Imaging Core Lab

The Central Imaging Core Lab will be coordinated by Dr. Paolo Remida, and will involve clinicians with long-standing expertise in assessing neuroradiological and neurointerventional data in stroke patients. See Chapter 10.2 for more details.

2 SCIENTIFIC JUSTIFICATION

2.1 The current state of knowledge - Rationale

Acute ischemic stroke (AIS) is one of the major causes of death and dependency worldwide. **Reperfusion therapies**, i.e. intravenous thrombolysis (IVT) and mechanical thrombectomy (MT), are highly effective and have become the standard of care.[1] However, their benefit depends on the subject-specific time window of the still salvageable ischemic brain tissue (i.e. the **ischemic penumbra**), whose evolution to irreversible infarction depends on the residual blood flow provided by **cerebral collaterals**. Over half of patients currently treated by MT remain disabled despite successful reperfusion.[2] The degree of collateral blood flow predicts successful versus futile reperfusion.

Investigating, developing and implementing a **collateral therapeutic** is a major objective in stroke research. The aim is to increase the amount of potentially salvageable penumbral tissue and expand the tissue time window, thus increasing the efficacy of reperfusion therapies and improving clinical outcomes. Several strategies have been proposed to boost collaterals in AIS, including partial aortic occlusion[3], external counterpulsation[4], sphenopalatine ganglion stimulation[5], high-dose albumin[6] and induced hypertension.[7] None has been currently validated.

Head down tilt 15° (HDT15) is a positional therapy consisting of tilting the patient with the head 15 degrees below the rest of the body. Experimental studies from an Italian-French group (Dr. Simone Beretta, Fondazione IRCCS San Gerardo dei Tintori and Prof. Tae-Hee Cho, Hospices Civils de Lyon) showed that HDT15 increased cerebral blood flow and improved functional outcome and infarct volume in randomised rats with middle cerebral artery (MCA) occlusion followed by reperfusion.[8,9] Subsequent perfusion MRI experiments using the same stroke model by our group confirmed that HDT15 application for 60 minutes significantly increases collateral flow in the ischemic area (results submitted to Stroke).

There is no consensus in current clinical practice regarding the most appropriate head position for AIS patients. The sitting position at +30° is the most common. An international cluster-randomised trial, HeadPoST, randomised over 11000 patients with acute stroke (85% ischemic) to either a lying-flat position or a sitting-up position (head elevated to at least 30°), maintained for 24 hours. No difference in the primary efficacy outcome (disability at 90 days measured with the modified Rankin Scale (mRS)), mortality, or rates of other serious adverse events, including pneumonia, were observed.[10] However, the HeadPoST trial primarily targeted patients with mild symptoms without a large vessel occlusion (LVO), who were randomised beyond the usual time window of reperfusion therapies. One retrospective study (pre-thrombectomy era) compared two cohorts of AIS patients with a LVO: patients with a standard position (0 to 30°; N=119), versus those with a HDT15 position (0 to -15°; N=90).[11] The results suggested that HDT15 promotes neurological improvement compared to the standard position. No difference in serious adverse events was observed between the two cohorts.

Thus, the available clinical evidence raised no safety concern for HDT15 in AIS patients, but data on its efficacy, notably among AIS patients treated by MT, remains insufficient. HDT15, for its simplicity, low cost and feasibility, might be an optimal collateral therapeutic candidate

to prolong the survival of the ischemic penumbra and improve the clinical benefit from reperfusion therapies with disability reduction. HDT15 is readily feasible by Emergency Services in the prehospital phase of AIS, before reperfusion therapies.

The DOWN-SUITE study will be the first multicenter, randomised, controlled, open-label clinical trial with blinded outcome assessment comparing collateral status in patients with AIS treated with an in-hospital application of HDT15 versus usual positioning before MT. The duration of HDT15 application (approximately 60-90 minutes) is expected to be long enough to detect significant changes in cerebral hemodynamics. Building from preclinical experiments on rodent and non-human primate stroke models[12] carried out by our French-Italian group, it will provide for the first time the translation of HDT15 efficacy on cerebral hemodynamics and clinical outcome from animal models to AIS patients. No therapeutic intervention is currently available to enhance collaterals in AIS.

The DOWN-SUITE trial will provide robust, high-quality evidence on the safety, feasibility and efficacy of HDT15 as a low-cost collateral therapeutic for AIS.

2.2 Research hypothesis

We hypothesise that HDT15 (Head down tilt 15°), applied in AIS patients with an LVO, will improve collateral circulation, prolong the survival of the ischemic penumbra and improve the clinical benefit from MT compared with standard of care (usual positioning: 0° to +30°).

2.3 Justification of the methodological choices

We aim to perform a prospective, multicenter, proof of concept, randomised, controlled, open-label study. As a double-blind is not possible, a blinded central imaging core lab, whose members will be unaware of the procedure assignments, will assess all imaging outcomes, including the primary efficacy outcome.

2.4 Target population

This study will involve adult patients who are eligible for MT and who have AIS due to left or right MCA occlusion (M1 segment). The selection criteria for participants are detailed in paragraph 5.

2.5 Benefit/risk ratio

Benefits:
We hypothesised that HDT15 would improve cerebral collaterals in patients randomised in the intervention group, which could subsequently reduce the ischemic injury and improve the clinical outcome. DOWN-SUITE aims to validate this hypothesis. Patients in the control group will expect no personal benefit or harm from participating in the trial as they will receive the standard recommended care.

Risks and constraints:
No harm from participating in DOWN-SUITE is expected for patients in the intervention or control groups. Previous clinical data on HDT15 raised no safety concerns.[11] No difference in post-stroke complications, including cerebral oedema or haemorrhage, pneumonia or

mortality, was observed in a retrospective observational study. HDT15 is not expected to interfere with standard care, including the procedural steps of MT. Mild discomfort related to the tilted position may occur in some patients. The treating physician will continuously monitor all patients during the entire duration of the HDT15 application. No additional risk is expected in patients in the control group, as they will receive standard care.

Overall, this study's benefit/risk ratio is considered very favourable.

2.6 Expected benefits

This study could potentially establish HDT15 as the first evidence-based collateral therapeutic for AIS. No therapeutic intervention is currently available to enhance collaterals in the acute phase of ischemic stroke. Such a collateral-enhancing therapy is necessary to expand the time window and increase the probability of successful reperfusion with IVT and MT, resulting in better clinical outcomes.

The results of the DOWN-SUITE trial will pave the way for larger randomised controlled trials of HDT15 in a wider stroke patient population, such as patients transferred from spoke hospitals to hub stroke centers to receive thrombectomy and unselected patients with suspected AIS in the prehospital setting.

The ultimate goal of this project will be the implementation of HDT15 as a practice-changing, standard-of-care emergency treatment of hyperacute stroke, easily implementable at a global level, with a revolutionary effect in the stroke care system and a positive impact on stroke outcome, with reduced costs of hospitalisation, long-term care and disability. With a positive result, HDT15 could be integrated into the international AIS management guidelines.

3 OBJECTIVES OF THE RESEARCH

3.1 Main objective

To assess whether in-hospital application of HDT15 is effective for improving cerebral collateral flow in patients with AIS due to the occlusion of the proximal segment (M1) of the middle cerebral artery treated with MT.

3.2 Secondary objective

The secondary objectives of the present study are:

- Assessment of the **feasibility:** proportion of those who are able to maintain HDT15 position during the MT procedure; time delay between hospital admission and arterial puncture for MT in both the intervention and control groups;
- **Safety:** physiological parameters (blood pressure, oxygen saturation) from hospital admission to the end of MT; vomiting from neuroimaging to the end of MT; neurological deterioration within 24±12 hours; occurrence of symptomatic intracranial haemorrhage (SICH) within 24±12 hours (see definition in paragraph 4.3.2); occurrence of pneumonia with the first 72 hours; these safety outcomes will be assessed in both the intervention and control groups
- **Clinical efficacy:** neurological outcome at 3 months in the HDT15 compared to the control group, assessed with the modified Rankin Scale (mRS).

4 RESEARCH DESIGN

4.1 Type of study

The DOWN-SUITE trial is a prospective, multicenter, proof of concept, randomised, controlled, open label, phase 2a/b clinical trial with blinded outcome assessment, comparing cerebral collateral status in patients with AIS due to M1 occlusion treated with in-hospital application of HDT15 versus usual positioning (0° to +30°), before and during MT.

4.2 Method for randomisation

Central randomisation will be performed by an online, secure, software application (Sealed Envelope; https://www.sealedenvelope.com) . The group of intervention will be automatically assigned following a pre-established randomisation list completed by the statistician of the study. The allocation ratio between the HDT15 (intervention) and usual position (control) groups will be 1:1. Randomisation will be stratified by site. To take into account the inter-individual heterogeneity in baseline collateral status, randomisation will also be stratified according to score of the National Institute of Health stroke scale (NIHSS) at presentation in the Emergency Room (dichotomised 0 to 5 versus 6 or more), which will be assessed before treatment allocation. Patients with NIHSS 0 to 5 will be considered as having a “minor stroke” despite a M1 occlusion, which will be considered as a surrogate marker of good baseline collaterals.

4.3 Endpoints

4.3.1 Primary endpoint

The **primary endpoint** will be the achievement of **good collateral status**, i.e. grade 3 or 4 on the American Society of Interventional and Therapeutic Neuroradiology/Society of Interventional Radiology (ASITN/SIR) collateral scale, in the HDT15 versus control group, as seen at the beginning of MT.[15] The ASITN/SIR collateral grade will be assessed by a blinded central imaging core lab from the pretreatment diagnostic angiographic runs, routinely performed as the first procedural step of MT. Patients achieving complete or partial recanalization (spontaneous or rt-PA-induced) at the first angiographic assessment during MT will be excluded from the primary outcome analysis, since ASITN/SIR score could not be assigned. Details regarding the assessment of the primary endpoint are available in **paragraph 10.2.2.**

4.3.2 Secondary endpoints

Secondary endpoints will further assess:

i. **feasibility** outcomes:
   - proportion of patients randomised to the intervention group who are able to maintain HDT15 during the entire MT procedure, encompassing patient tolerance, the ability to perform MT at -10° to -15° tilt and any other reasons for premature treatment termination;
   - hospital admission-to-arterial access time (i.e. time to arterial puncture);

ii. **safety** outcomes:
   - Mean values of systolic blood pressure, diastolic blood pressure and oxygen saturation from hospital admission to the end of MT; these physiological parameters will be monitored at entry in the Emergency Room, at entry in the angio suite and every 15 minutes from the start to the end of MT
   - Proportion of patients presenting one or more episodes of vomiting from randomisation to the completion of MT
   - Proportion of patients who had increase of ≥4 points on the NIHSS score within 24±12 hours of the randomisation;
   - Proportion of patients who had SICH per SITS-MOST definition within 24±12 hours: type 2 parenchymal hematoma with neurological deterioration of ≥4 points on the NIHSS;
   - proportion of patients with pneumonia within the first 72 hours after randomisation;
   - Safety data, including serious and non-serious adverse events, as well as reasons for HDT15 discontinuation (e.g. discomfort, vomiting, neurological worsening, headache, respiratory distress) and technical challenges related to MT will be reported for both treatment arms;

iii. **efficacy** outcomes:

- functional outcome at 3 months, assessed with the ordinal score on the modified Rankin scale (shift across outcomes on the mRS between groups);
- early neurological improvement before MT, at 24±12 hours and at 7 days or discharge (whichever comes first), defined as percent change in NIHSS score [(Admission NIHSS — time-point NIHSS) × 100/Admission NIHSS].

5 ELIGIBILITY CRITERIA

5.1 Inclusion criteria

- Adults (≥ 18 years old)
- Acute ischemic stroke due to left or right proximal MCA occlusion (M1 segment; excluding concomitant occlusion of the ipsilateral internal carotid artery and/or not visible A1 segment of the ipsilateral anterior cerebral artery)
- Decision to treat with mechanical thrombectomy (with or without intravenous thrombolysis)
- Informed consent obtained from patient or patient's legal representative, or emergency consent procedure

5.2 Exclusion criteria

- Impaired consciousness, defined as NIHSS score of 2 or 3 of the item 1a (level of consciousness): not alert, requires repeated stimulation or unresponsive.
- Vomiting upon stroke onset.
- History of glaucoma.
- History or imaging findings of intracranial hypertension of any aetiology
- Major breath disorders, defined as follows:
    - oxygen saturation ≤92% in room air at admission
    - severe chronic obstructive pulmonary disease (COPD) treated with long-term oxygen therapy.
    - severe heart failure with NYHA class 3 or 4 (breathlessness during ordinary physical activity or at rest).
- Severe obesity, defined as body mass index (BMI) > 35.
- Patients participating in another interventional trial that would interfere with this study.
- Female patients who are pregnant

5.3 Premature study exit criteria

The criterion for premature study exit for a patient is the withdrawal of consent.
Early withdrawal will not affect standard of medical care. Patients with premature exit from the study will not be replaced.

5.4 Criteria for discontinuation of study procedure

HDT15 will be discontinued in cases of patient-reported intolerable discomfort, observed severe discomfort in non-communicative patients, or at the treating physician's discretion for clinical reasons (e.g., vomiting). All reasons for discontinuation will be recorded and reported as safety data.

5.5 Recruitment procedures

Patients will be included at admission in the stroke emergency room of academic hospitals, each including a comprehensive high-volume stroke center. The DOWN-SUITE will start in three Italian centers, but an extension to other clinical sites may be considered, depending on available funding and the progress of the study.

After the baseline neuroimaging, including intracranial vascular imaging, the study investigator will check all inclusion and exclusion criteria and assess the patient's eligibility for the study. After the consent process and randomisation, the intervention will be performed as soon as possible in all patients.

6 EXPERIMENTAL STRATEGIES

6.1 Study strategy: experimental group

6.1.1 Description of the strategy

HDT15 will be applied in the intervention group in 2 different settings:
- in the Emergency Room, by tilting the stretcher to lower the head by -10° to -15° relative to the body of the patient; the degree of tilting will be checked using a dedicated digital inclinometer or a mobile phone app
- in the Angiography Suite, by tilting the angiography table to lower the head by -10° to -15° relative to the body of the patient, depending on the actual technology of the angiography system of each clinical site; the degree of tilting will be automatically checked using the angiography system

HDT15 will start as soon as possible following randomisation in the Emergency Room (i.e. after vascular neuroimaging), and will be maintained during the transfer to the Angiography Suite, as well as during the entire thrombectomy procedure: i.e. HDT15 will end after the completion of MT, when the patient is transferred from the angiography table to her/his bed in a standard flat position.

Application of HDT15 will not delay usual care. The standard in-hospital patient pathway, including transfer from neuroimaging room to the angiography suite and the required procedural steps of MT, will allow sufficient time for the application of HDT15. HDT15 duration is expected to be at least 30 minutes (estimated time 30 to 90 minutes), from start to the assessment of the primary efficacy endpoint.

The actual degree of HDT15 will be between -10° to -15°, depending on the ER stretchers and the angiographic system of each clinical site.

Despite conscious sedation being the preferred approach for MT in participating centers, for patients needing intubation, the HOB will be briefly set to 0° during tube placement to ensure safety, with immediate repositioning to the assigned position (HDT15: -10° to -15°; control: 0° to +30°) post-intubation to maintain protocol adherence.

6.1.2 Justification of the chosen strategy

No collateral therapeutic has been currently validated (see paragraph 2.1). There is no consensus in current clinical practice regarding the most appropriate head position for AIS patients. Both experimental and available clinical data on HDT15 suggest its potential benefit in boosting the collateral circulation with a very favorable safety profile. Moreover, the simplicity and low cost of HDT15 will have profound implications on global AIS management if this strategy is validated.

6.1.3 Description of contraindications

There are no contraindications apart from those listed in the exclusion criteria list (See paragraph 5.2).

6.2 Strategy of comparison : control group

6.2.1 Description of the strategy

Patients randomised in the control group will be maintained in the usual position during the emergency room phase (0° to +30°) and on the angiography table (0°), according to standard practice. MT will be performed as per usual care.

6.2.2 Justification of the chosen strategy

Patients in the control group will be managed according to standard practice.

6.2.3 Description of contraindications

There are no contraindications apart from those listed in the exclusion criteria list (See paragraph 5.2).

6.3 Blinding

6.3.1 Organisation

Double-blinding of the study intervention is not possible. The Neurologist and Neurointerventionist in charge of the patient will remain in close proximity of the patient during HDT15 application, and cannot be blinded. Similarly, the patient cannot be blinded to the treatment allocation, given the nature of the procedure (HDT15 or flat position). Thus, this study is an open-label trial but with a blinded assessment of all endpoints, including the primary outcome, which will be assessed by a central imaging core lab.

6.3.2 Unblinding

No unblinding procedure is planned for this study.

6.4 Permitted and prohibited associated treatments

All treatments part of the standard management of AIS and/or deemed necessary by the treating medical team will be allowed during the study. The only prohibited treatment during the study will be experimental drugs or devices evaluated in other ongoing clinical trials.

7 GENERAL ORGANISATION

7.1 Schedule of the study

Duration of the inclusion period: 15 months
Duration of participation for each patient/healthy subject: 3 months ± 15 days
Total study duration: 18 months ± 15 days
Start of inclusions: Third quarter of 2024

The start of the study is defined by the first visit of the first patient (usually corresponds to the signing of the consent form). The end of the study is defined as the last visit of the last patient.

As soon as the first patient is included, the Sponsor must inform the competent Ethics Committee without delay of the effective starting date of the study (effective starting date = date of signature of the consent/date of receipt of the non-objection of the first person who takes part in the research).

7.2 *Summary table*

Time point ***Actions***	***Visit 1*** *Inclusion*	***Visit 2***	***Visit 3***	***Visit 4*** *Clinical visit or Phone Call*
	Hospital admission	***Day 1*** *24±12 hours*	***Day 7±2*** *or hospital discharge*	***Day 90±15*** *long-term follow-up*
Eligibility screening	X			
Informed consent or emergency process	X			
Brain neuroimaging[1]	X	X		
Randomisation	X			
Study intervention (HDT15 or control)	X			
Angiography / thrombectomy: ASITN/SIR collateral scale, TICI recanalisation score	X			
Demographic data	X			
Medical history	X			
mRS score	X *pre-stroke*			X[5]
Weight and height	X			
ECG	X			
Routine biological analysis[2]	X	X		
Physiological parameters[3]	X	X		
NIHSS score[4]	X	X	X	
TOAST classification[5]				X
AE and SAE records[5]	X	X	X	X
Any reasons for HDT15 discontinuation	X			

[1] Including intracranial vascular imaging, may be performed using either CT or MRI
[2] Routine biological analyses: complete blood count, glycaemia, sodium, potassium hepatic profile, serum creatinine and lipid profile (at 24h only), C-reactive protein.
[3]Physiological parameters: blood pressure, heart rate, oxygen saturation at inclusion (at entry in the Emergency Room; at entry in the angio suite; every 15 minutes from the start to the end of MT) and at 24 hours
[4]NIHSS score at admission will be measured both before enrollment and immediately before MT.
[5]mRS, AE/SAE and TOAST classification at 90 days may be collected via phone by a trained coordinator if in-person visit is not feasible.

7.3 *Conduct of the study*

7.3.1 *Screening - Information and informed consent*

Patients eligible for enrolment will be identified at admission, once routine neuroimaging has been completed. All patients with AIS due to left or right M1 occlusion will be screened for participation. Inclusion and exclusion criteria will be assessed based on information available at admission.
The investigator's decision to enroll a patient must be based on an appropriate benefit–risk balance. The patient, or his/her legally authorized representative, will be fully and fairly informed, in understandable terms, about the objectives and constraints of the study, the possible risks, the required monitoring and safety measures, and the right to refuse participation or to withdraw at any time. This information is provided in an information and consent form, and written informed consent will be obtained by the investigator prior to inclusion.

If the patient is unable to provide informed consent and no legally authorized representative is available at the time of enrollment, an emergency consent procedure (EU 536/2014, section 36; 2025 AIFA guidelines) will be applied. Considering the AIS emergency situation, the time-dependent nature of the experimental treatment, and the favorable safety profile of HDT15, the investigator will inform the patient's next of kin, if available, about the study. In such cases, the investigator documents in the medical record that the emergency consent procedure has been applied and signs the relevant documentation. The patient or his/her legal representative must then provide full written informed consent as soon as the patient's clinical condition allows it (deferred consent). Emergency consent must always be documented before performing any clinical or paraclinical examination required by the study.

In all cases, the patient's assent will be explored. If the investigator has reason to believe that the patient would refuse the procedure, the treatment will not be administered.
If the patient, or the legally authorized representative (or the next of kin in case of emergency consent), decides to withdraw from the trial, the patient will be discontinued from the study.

In all cases, the investigator must record the progress of the inclusion in the medical file, and written informed consent must be obtained as soon as the patient's clinical condition allows it.

7.3.2 *Inclusion visit / Randomization*

Central randomisation will be performed by an online software application (https://www.sealedenvelope.com), just after the written consent or emergency form is signed and as soon as possible after admission neuroimaging has been obtained.

The intervention will be performed as soon as possible after the randomisation. In-hospital application of HDT15 will start immediately after randomisation and will last until the completion of MT. In the control group, MT will be performed in the standard flat position. Patient will undergo reperfusion according to international recommendations.
The degree of HDT (-10° to -15°) and control group (0° to +30°) will be recorded.

Demographic information and medical history, the site of intracranial occlusion, the administration of intravenous thrombolysis (rt-PA) or not, the method of anesthesia used during MT (i.e. local anesthesia only, conscious sedation or general anesthesia), the blood pressure monitoring as well as the technical aspects of the endovascular procedure (concomitant drugs, thrombectomy devices, degree of recanalisation using the TICI score) will be reported in medical records.

Clinical study visit at admission will consists of a clinical (including weight, height, blood pressure, heart rate and temperature) and neurological examination (NIHSS and pre-stroke mRS score), an assessment of serious adverse events, a 12-lead electrocardiogram and routine blood chemistry tests.

The admission neuroimaging will include either CT or MRI, including intracranial vascular imaging, digital subtraction angiography (MT procedure) and follow-up (24 hours) neuroimaging.

7.3.3 Follow-up visits

- **Visit 2 – Day 1 (24 ± 12 hours)**

Clinical study visit at 24 hours, performed by a neurologist, will consists of a clinical and neurological examination (NIHSS score), an assessment of serious adverse events, and routine blood chemistry tests (including complete blood count, sodium, potassium, glycemia, Hba1c, total bilirubin, alkaline phosphatase, alanine et aspartate transaminase, gamma-glutamyl transferase, serum creatinine, troponin and C-reactive protein and lipid profile).

A second neuroimaging (either brain CT or MRI), part of the routine follow-up of acute ischemic stroke patients, will be performed to control the evolution of stroke and detect early complications (such as SICH).

- **Visit 3 – Day 7 ± 2 days or hospital discharge**

At Day seven or at hospital discharge (whichever comes first), the clinical study visit, performed by a neurologist, will consists of a neurological (NIHSS score) examination and an assessment of serious adverse events.

- **Visit 4 – Day 90 ± 15 days**

This will be the last visit of this trial. Clinical study visit by the neurologist at 90 days will consist of a standardised assessment of neurological disability (mRS score), the evaluation of stroke etiology (TOAST score), and an assessment of serious adverse events.

If a clinical visit can't be performed, a phone call will be scheduled in order to collect the mRS score and the serious adverse events.

7.3.4 Imaging tests required for the project

Routine neuroimaging (CT or MRI scan at admission, digital subtraction angiography during MT and CT or MRI scan at 24 hours) will be retrieved, anonymised and stored via a dedicated secure platform (XNAT; https://www.xnat.org) for the blinded evaluation of imaging

endpoints. This image management will be carried out by the Sponsor under the responsibility of Dr. Paolo Remida.

Imaging data centralisation will be performed continuously during the study. Imaging will be reviewed by the Central Imaging Core Lab by batch in order to obtain all the blinded assessments at the end of the study. Imaging data, produced by the Central Imaging Core Lab, will be stored in the study database. Futher details are available in paragraph 10.2.2.

7.4 Temporary or permanent cessation rules

- *Terminating a person's participation in research:*

Subjects may withdraw their consent and ask to leave the study at any time and for any reason. In the event of premature withdrawal, the investigator should document the reasons as fully as possible. If a subject is lost to follow-up, the investigator will make every effort to reconnect with the person and, if possible, collect the reason for the loss of contact. In the event of withdrawal of consent, the data collected up to the date of withdrawal will be analysed. These patients will not be replaced. For patients included with the emergency process and for whom written deferred consent is asked after randomisation, in case of refusal, the patient data will be analysed until "refusal of follow-up" date unless the patient disagrees. These patients will not be replaced.

- *Terminating part or all of the research:*

The study may be prematurely terminated in the event of unexpected, serious adverse events requiring a review of the strategy profile. Similarly, unforeseen events or new information about the method of investigation, which make it unlikely that the objectives of the study will be achieved, may lead the sponsor to prematurely terminate the study. The University of Milano-Bicocca reserves the right to discontinue the study at any time if it is determined that the inclusion objectives are not being met. In case of premature termination of the study for safety reasons, the information will be transmitted by the sponsor without delay to the competent Ethics Committee.

8 SAFETY ASSESSMENT

8.1 Definitions

According to Good Clinical Practice guidelines E6(R2).

8.1.1 Adverse event (AE)

An adverse event (AE) is any untoward medical occurrence in a patient or subject which does not necessarily have a causal relationship with the research involving a human person.

8.1.2 Serious adverse event (SAE) or reaction

Any adverse event or reaction:
- which results in **death**; or
- that is **life-threatening** for any person who participates in the clinical trial; or
- which requires **hospitalisation or prolongation of existing hospitalisation**; or
- which results in in **persistent or significant disability or incapacity**; or
- which results in a **congenital anomaly or birth defect**; or
- any **other important medical events** that do not meet the qualifications listed above:
  - but which may be considered **“potentially serious” or “medically relevant” in the judgment of the investigator**;
  - or **an event requiring medical intervention** to prevent one of the above characteristics/consequences

The term “life-threatening” is reserved for an immediate life-threatening situation at the time of the adverse event, regardless of the consequences of corrective or palliative therapy.

Some circumstances requiring hospitalisation do not fall under the severity criterion.
Refer to the paragraph 8.2.4 for **serious adverse events not requiring immediate notification to the sponsor, since they are expected as part of the natural history of the disease under study (acute ischemic stroke).**

8.1.3 Adverse reaction

An adverse reaction is any untoward medical occurrence in a patient or subject causally related to the procedure under investigation (HDT15), involving a human person.

8.1.4 Unexpected adverse reaction

An adverse reaction whose nature, severity, frequency or outcome is not consistent with the safety information reported in the available clinical studies on lower head positioning in acute ischemic stroke.

8.1.5 New issue

Any new information that may lead to a reassessment of the risk-benefit balance of the procedure under investigation (HDT15), to changes in the use of the procedure, in the conduct of the research, or in the documentation of the research, or to the suspension or discontinuation or modification of the research protocol or similar research.

8.2 *Investigator's responsibilities*

8.2.1 *Procedures for detecting and collecting adverse events*

From the first visit (hospital admission) until the end of the study and until their resolution, all adverse events and special situation, except those not requiring collection mentioned in paragraph 8.2.4, must be sought, reported and recorded, processed and evaluated, in the case report form. The following events are not considered to be adverse events (and therefore should not be collected in the eCRF):

- admission for social or administrative reasons;
- protocol-defined hospitalisation;
- a visit to a day hospital scheduled as part of the follow-up of the pathology studied or of an intercurrent disease already known at inclusion.

The **intensity of adverse events will be graded** as follows:

- mild (grade 1): no interference with the patient's daily activity
- moderate (grade 2): moderate interference with the patient's daily activity but still acceptable
- severe (grade 3): significant interference with the patient's daily activity and unacceptable
- life-threatening (grade 4)
- death (grade 5)

All adverse events should be graded. All **life-threatening and fatal** adverse events **(grade 4 or higher) are considered SEVERE and should be reported to the sponsor without delay** *(unless described in paragraph 8.2 4 as not requiring prompt reporting to the sponsor).*

- each grade of adverse event should be reported separately in the eCRF (one line per grade if the grade changes for the same AE);
- the end date of a SAE due to hospitalisation or prolongation of hospitalisation or life-threatening event is the earliest date occurring between the date of end of hospitalisation or the date of return to the previous grade before the SAE occurs, unless the investigator considers another date to be clinically relevant;
- the end date of an SAE due to a medically significant event is the date of return to the previous grade;
- if an AE involves biological parameters, the start and end date should be based on the date of blood sampling and not the date of the laboratory report.

8.2.2 *Immediate notification of Serious Adverse Events (SAEs) to the sponsor*

The investigator assesses each adverse event in terms of its severity.
The investigator should **report all serious adverse events occurring in the trial to the Data and Safety Monitoring Board (DSMB) without delay, except those identified in the protocol as not requiring immediate reporting** (see 8.2.4).
This initial notification is reported and must be followed by a detailed additional report(s) within 8 days of the first notification.
The members of the DSMB may notify the Sponsor at their discretion.

The investigator should document the event to the best of their ability (through copies of laboratory results or test or hospital reports documenting the serious event, including relevant negative results, **without omitting to anonymise these documents** and to record the patient's number and code), the **medical diagnosis** and to establish **a causal relationship** between the serious adverse event and the medical device(s) and the action taken.

The investigator should follow the patient who has experienced an SAE until resolution, stabilisation at a level acceptable to the investigator or return to baseline, even if the patient has been discharged from the trial, and inform the sponsor.

8.2.3 Assessment of the causality of AEs

The Investigator should assess the causal relationship of adverse events to the research. **Causality is binary (reasonably related / unrelated).**

8.2.4 Restriction of AEs/SAEs and reporting period of AEs/SAEs

Given that the study is being conducted in patients with acute ischemic stroke, restrictions on the notification of SAEs to the safety sector of clinical trials have been put in place to account for adverse events which are expected as part of the natural history of the patient population under study.

Restriction of SAEs (SAE notification form not to be immediately sent to the DSMB but only to be collected in the CRF at the latest at the end of the study participation):

- *Adverse events related to the procedure (HDT15) under study:*
  There is no expected serious adverse event related to HDT15 in the intervention group of patients.

- *Adverse events related to the disease under study (acute ischemic stroke):*
  - ⇨ Death (as it is expected from epidemiological data),
  - ⇨ Hemorrhagic transformation of the ischemic lesion,
  - ⇨ Stroke recurrence,
  - ⇨ Malignant cerebral infarction,
  - ⇨ Early neurological deterioration,
  - ⇨ Cardio circulatory arrest,
  - ⇨ Cardiogenic shock,
  - ⇨ Myocardial infarction,
  - ⇨ Heart failure,
  - ⇨ Acute pulmonary edema,
  - ⇨ Pulmonary embolism,
  - ⇨ Pneumonia,
  - ⇨ Urinary tract infection,
  - ⇨ Atrial fibrillation,
  - ⇨ Severe arterial hypertension, defined as blood pressure over 180/110 mmHg.

- *Adverse events related to MT and/or intravenous thrombolysis:*
  - ⇨ Arterial dissection,

- ⇨ Artery perforation,
- ⇨ Vasospasm,
- ⇨ Stent occlusion,
- ⇨ Intracranial hemorrhage (sICH), either symptomatic or not,
- ⇨ Cerebral or systemic embolism,
- ⇨ Facial angioedema,

The investigator should notify the sponsor any serious adverse events, not included in the above specified ones, without delay:

- *FROM THE* PATIENT'S *INCLUSION (date of signature of the 1st consent)*
- Until the *END OF THE PATIENT'S PARTICIPATION*
- No time limit for serious adverse events related to the research (e.g. cancers, congenital malformations occurring in the long term after exposure to the investigational medicinal product...).

8.3 Responsibilities of the sponsor

8.3.1 Declaration to the competent authorities

The sponsor should expedite the reporting to all concerned investigator(s)/institutions(s), to the Ethics Committee, where required, and to the regulatory authority of all adverse drug reactions that are both serious and unexpected. Such expedited reports should comply with the applicable regulatory requirement(s) and with the ICH Guideline for Clinical Safety Data Management: Definitions and Standards for Expedited Reporting.

8.4 Data Safety Monitoring Board

The Data and Safety Monitoring Board will provide expertise by periodical review and evaluation of the accumulated study data for participant safety, study conduct and progress; it will also provide recommendations to the Steering Committe concerning the continuation, modification, or termination of the trial.

9 STATISTICAL ASPECTS

9.1 Number of subjects needed

Considering previous data on distribution of acute stroke patients across the ASITN/SIR collateral scale[13], a sample size of 118 patients (59 patients per group) should yield 80% power to detect a between group difference of 25% (60% in experimental group versus 35% in control group) for the primary endpoint, which is considered of substantial clinical significance, with a bilateral alpha risk of 0.05.

9.2 Analysis population

The intention-to-treat population is defined as all patients included in the study according to the group allocated at randomisation, regardless of eligibility criteria and amount of

intervention received, whether evaluable or non-evaluable for the endpoints. The description of the population at inclusion will be on an intention-to-treat basis.

Analyses will be conducted on a modified intention-to-treat basis (after exclusion of patients achieving complete or partial recanalization at the first angiographic assessment during MT), with a secondary per-protocol analysis of the primary endpoint.

The per protocol population is defined as the modified intention-to-treat population from which patients with major protocol deviations will be excluded. Major deviations will be reviewed in the blind review, during which additional blinded cases may be identified in the intervention group. These will be specified in the statistical analysis plan. Patients will be considered in the actual group. A secondary analysis of the primary endpoint will be performed per-protocol.

The safety population is defined as the population of patients who received the actual intervention. The safety and tolerability criteria will be assessed according to this population.

9.3 Statistical methods

9.3.1 General considerations

The quantitative variables will be described by the following parameters: number of patients, number of missing values, mean, standard deviation (SD), median, first and third quartiles (Q1 and Q3), minimum and maximum. Categories could be defined if applicable using a cut-off threshold from literature or quantiles.

The qualitative variables will be described by the following parameters: number of patients, number of missing values, frequency and percentage of each modality (missing values will not be included in the denominator used for frequency computation).

The analyses will be performed using the R and SAS software. They will be performed by the biostatistics unit of the University of Milano-Bicocca.

After the end of the statistical analysis of the DOWN-SUITE study, a meta-analysis could be carried out with the French partner (Prof. Tae-Hee Cho, Hospices Civils de Lyon), who applied for funding for a similar study (DOWN-SUITE-Fr, the result of which is pending).

9.3.2 Primary endpoint

The analysis of the primary endpoint will be performed by a mixed effects logistic regression model. It will take into account as explanatory variables the group of intervention, as well as the stratification factors (the site as random effects and the baseline NIHSS score [dichotomised 0 to 5 versus 6 or more] as fixed effect). The effect of the intervention on the occurrence of good collaterals will be assessed by a Wald test and quantified through the adjusted odds ratio with the associated 95% confidence interval.

The agreement between readers will be estimated using the Cohen’s kappa or the weighted Cohen’s kappa coefficient for the dichotomised ASITN/SIR collateral score (good collaterals: score 3 to 4; poor collaterals: score 0 to 2) and the ASITN/SIR collateral ordinal score,

respectively. Coefficients of ≤0.20, 0.21-0.40, 0.41-0.60, 0.61-0.80 and >0.80 indicate poor, fair, moderate, good and excellent agreement, respectively.

A pre-planned secondary analysis will evaluate the effect of HOB positioning on the primary endpoint (ASITN/SIR collateral score, grade 3–4) across all patients, treating HOB angle as a continuous variable calculated as a time-weighted average (integrating angle and duration per patient to account for within-patient variations, including HDT15 patients requiring standard positioning due to intolerance). In particular, for each patient, the time-weighted average lower HOB exposure" will be calculated as follows, with higher values representing greater exposure to lower head positions:

$$time\text{-}weighted\ lower\ HOB\ exposure = \left(\sum \frac{(30° - HOB\ angle + 1) \times duration}{total\ duration}\right)$$

This analysis will use a mixed-effects logistic regression model, adjusted for stratification factors (site as random effect, baseline NIHSS [0–5 vs. ≥6] as fixed effect) and covariates (baseline collateral status on vascular neuroimaging at admission; intravenous rt-PA treatment), reporting adjusted odds ratios with 95% confidence intervals.

Pre-specified sensitivity analyses will include: (1) per-protocol vs. modified intention-to-treat analysis; (2) inclusion of only those patients with baseline NIHSS ≥6; (3) exclusion of HDT15 patients with tilting duration <50% of the time from randomization to angiographic collateral assessment; and (4) comparison of control arm subgroups (0° to +5° vs. +6° to +30°) to HDT15. The number of patients achieving complete or partial recanalization (spontaneous or rt-PA-induced) at the first angiographic assessment during MT will be reported for both arms.

9.3.3 Secondary endpoints

i. **feasibility** outcomes:
   - The proportion of patients randomised to the intervention group who are able to maintain HDT15 during the entire MT procedure will be calculated with their 95% confidence interval.
   - The hospital admission-to-arterial access time (i.e. time to arterial puncture) will be described by median, first and third quartiles (Q1 and Q3) for each group.

ii. **safety** outcomes:
   - Mean values of systolic blood pressure, diastolic blood pressure and oxygen saturation at entry in the Emergency Room, at entry in the angio suite and every 15 minutes from the start to the end of MT; they will be treated as continuous variables and calculated as mean with their 95% confidence interval for each group.
   - Proportion of patients presenting one or more episodes of vomiting from randomisation to the completion of MT will be calculated with their 95% confidence interval for each group.
   - Reasons for HDT15 discontinuation (e.g., intolerable discomfort, observed severe discomfort in non-communicative patients, vomiting, neurological worsening,

headache, or respiratory distress), minor adverse events, and MT-related technical challenges will be recorded and reported as proportions with 95% confidence intervals.
- The proportions of patients who had an increase of ≥4 points on the NIHSS score within 24±12 hours of the randomisation will be calculated with their 95% confidence interval for each group.
- The proportions of patients who had SICH within 24±12 hours will be calculated with their 95% confidence interval for each group.
- The proportions of patients with pneumonia within the first 72 hours after randomisation will be calculated with their 95% confidence interval for each group.
- Safety data, including serious and non-serious adverse events, as well as reasons for HDT15 discontinuation (e.g. discomfort, vomiting, neurological worsening, headache, respiratory distress) and technical challenges related to MT will be reported for both treatment arms. Adverse events will be categorized according to the Medical Dictionary for Regulatory Activities (MedDRA) v28.0.

iii. **efficacy** outcomes:
- functional outcome at 3 months, assessed with the ordinal score on the modified Rankin scale (shift across outcomes on the mRS between groups).
- Percent change in NIHSS ([(Admission NIHSS – time-point NIHSS) × 100 / Admission NIHSS]) from admission to immediately pre-MT, at 24±12 hours, and at 7±2 days (or discharge), calculated with 95% confidence intervals.

The analysis of the effect of the group on the mRS at 3 months will be performed by the mixed effects ordinal logistic regression model. It will take into account as explanatory variables the group of intervention, as well as the stratification factors (the site and the baseline NIHSS score [dichotomised 0 to 5 versus 6 or more] as random effects). The effect of the intervention on the occurrence of good collaterals will be assessed by a Wald test and quantified through the adjusted common odds ratio with the associated 95% confidence interval.

9.4 Intermediate analyses

An interim analysis of the primary and secondary outcomes is planned 12 months after the start of recruitment. Based on the results of this analysis and the progress of enrolment, the Steering Committee may consider a reassessment of the sample size and a recalculation of the study's statistical power.

9.5 Method of taking missing data into account

Analyses will be performed on data available. There will be no imputation of missing data.

9.6 Managing changes to the analysis plan

A detailed statistical analysis plan will be drawn up before the database is frozen. It will take into account any changes in the protocol or unexpected events during the course of the study that have an impact on the analyses presented above. Planned analyses may be completed in line with the study objectives.

Any subsequent changes to the statistical analysis plan must be justified and will result in a new version of the document. These deviations from the analysis plan will be reported in the final report of the study. All documents will be kept in the study file.

10 CENTRAL IMAGING CORE LAB

10.1 Role

The Central Imaging Core Lab will be coordinated by **Dr. Paolo Remida**, and will involve clinicians with long-standing expertise in assessing neuroradiological and neurointerventional data in stroke patients.
The Central Imaging Core Lab will review all imaging data (admission neuroimaging, digital subtraction angiography, follow-up neuroimaging) and assess all imaging outcomes, including the primary outcome. The Core Lab will provide reference judgments in a blinded and standardised way for all imaging related endpoints. This process allows a harmonisation of all imaging outcome assessment, which significantly increases the reliability of the results of a study. The establishment of such a board is particularly useful in multicenter studies where medical practice may differ from centre to centre. The modalities of operation of this blinded central imaging core lab will be described in a charter at the beginning of research. Only the assessed imaging outcomes will be used for statistical analyses. The Central Imaging Core Lab will be headed by Dr. Paolo Remida and will include senior Neuroradiologists.

10.2 Technical aspects

The primary endpoint is the achievement of a good collateral status, defined as grade 3 or 4 on the ASITN/SIR scale. This scale is the widely accepted reference method for grading the collateral circulation in AIS patients, using the gold-standard method of digital subtraction angiography. The standardised technical aspects were detailed in the position paper of the ASITN/SIR[12], and are reproduced here:

Grade 0	No collaterals visible to the ischemic site
Grade 1	Slow collaterals to the periphery of the ischemic site with persistence of some of the defect
Grade 2	Rapid collaterals to the periphery of ischemic site with persistence of some of the defect and to only a portion of the ischemic territory
Grade 3	Collaterals with slow but complete angiographic blood flow of the ischemic bed by the late venous phase
Grade 4	Complete and rapid collateral blood flow to the vascular bed in the entire ischemic territory by retrograde perfusion

“Time of collateral filling” is somewhat subjective; however, it might be determined by counting the number of frames from contrast-agent filling of the petrous carotid artery (from the anterior circulation) and the proximal basilar artery (in the posterior circulation) to complete collateral filling (provided that the number of frames per second is known, so that the time for collateral filling can be calculated.) This is the n compared with time to normal filling of the nonoccluded hemisphere in the parenchymal phase of the angiogram. Slow collateral flow is defined, arbitrarily, as filling that is 2 seconds slower than the contralateral side. Rapid collateral flow is defined as filling that is within 2 seconds of the contralateral side. It is essential that the angiogram include both the arterial and venous phases of the injection to evaluate the collateral pathways.

In order to strictly follow the ASITN/SIR grading recommendations (detailed above), the acquisition parameters of the angiography runs for assessing the primary endpoint will be

specified and homogenised across all recruiting centers (anterior and lateral field of view, frame rate, inclusion of arterial and late venous phases).

The Central Imaging Core Lab will also evaluate the baseline neuroimaging and determine the pretreatment collateral status of all patients, using a dichotomised version of the criteria reported by Tan et al.[14] (collateral supply filling 0% to 50% versus 50% to 100% of the occluded MCA territory).

Two independent experts (Interventional Neuroradiologists), blind to treatment allocation and not involved in patient recruitment will independently grade all patients, thus allowing for the evaluation of inter-rater reproducibility. All discrepant cases will be resolved by an third independent expert, also blind to treatment allocation.

11 ACCESS RIGHTS TO SOURCE DATA AND DOCUMENTS

11.1 Access to data

In accordance with GCP:
- the sponsor is responsible for obtaining the agreement of all parties involved in the research to ensure direct access to all research sites, source data, source documents and reports for the purpose of quality control and audit by the sponsor;
- investigators will make available to the persons responsible for monitoring, quality control or auditing of research involving human subjects, the documents and individual data strictly necessary for this control, in accordance with the legislative and regulatory provisions in force.

11.2 Data collected

The health personal data collected are:
- Demographic data and medical history
- Intervention and medications data
- Clinical and neurological data (including scores)
- Imaging and biological data
- Length of stay in hospital
- Adverse events data

Theses data will be collected from the medical record, hospitalisation report and examination results (biological and imaging reports). Data collected from patients who left the study prematurely and accept their conservation will be analysed according to the study objectives.

11.3 Source documents

Source documents are defined as any original document or object that can be used to prove the existence or accuracy of a data or fact recorded during the clinical study. In the case of a hospital medical record, they will be kept for the duration of the patient's operational management, and then archived for 15 years from the date of the last patient's last visit.

In this study the source document could comprise:

- medical file;
- hospitalisation report;
- original copy of the biological examination results;
- imaging examination report ;
- data of the Central Imaging Core Lab.

11.4 Data privacy

The personal data object of the study must be treated in compliance with the European Regulation on the Protection of Personal Data (GDPR), the Legislative Decree 196/2003 and subsequent amendments and additions, and any other Italian law applicable to the protection of personal data (henceforth referred to as the "applicable data protection law").
Data will be accessible only to authorised personnel. Personal authorisation profiles will be created in order to regulate access to the database. In particular, only the clinical staff responsible for evaluating the subjects and the appointed staff responsible for entering the data will have access to personal identification data.
The sponsor will ensure that each person who takes part in the research has given written consent for access to their individual data which is strictly necessary for the quality control of the research.
Users authorised to use the eCRF will be able to access it only through the use of individual access credentials (username and password). The login credentials will be generated by the database administrator and communicated to the user via e-mail. The user must change the password on the first access. The password will need to be changed every 3 months.
Source data are hospital standard data, and are already treated according to privacy laws.
The data will be collected in pseudonymised form: for each subject an alpha numeric code (subject code) will be generated consisting of the center code, a spacer character and the subject number (e.g. CTR01-01) and the data will be stored electronically.
To keep track of the association between the medical record and the subject code, each center will compile a register which will contain: subject code, name and surname, date of birth and date of screening. This register, if in paper form, must be kept in a place with limited access and locked; if in electronic form, it must be kept in a separate, encrypted (AES-256 encryption) and password protected file; this register will never be shared with the Promoter, let alone with the database administrator and not even with the study statistician.
Data on eligible subjects not entering the study will be pseudonymised from start, since the relative list will contain only age (years), sex and reason for not entering the study.
Direct access to source documents will be allowed for monitoring, audits or inspections.

The project data can be accessed by interested European and extra-EU third parties for replication or further analysis only if given in a completely anonymous form. If the need arises to communicate data in pseudo-anonymous form, the patient will, first of all, be informed and specific informed consent will be provided. In the latter case, the data will not be sent without the patient's consent. Ad hoc Data Transfer Agreements will be established with the interested third parties.
Third parties in this project include academic institutions, research centers and non-profit associations (e.g. research consortia). Third parties in this project do not include profit associations (e.g. drug companies).

12 STUDY MONITORING, QUALITY CONTROL AND ASSURANCE

A Clinical Research Associate (CRA) mandated by the sponsor will ensure that the study is carried out correctly, that the data generated are collected in writing, documented, recorded and reported, in compliance with Good Clinical Practice as well as with the legislative and regulatory provisions in force.

The investigator and the members of their team agree to be available for Quality Control visits conducted at regular intervals by the CRA. During these visits, the following items may be reviewed according to the level of monitoring appropriate to the study and determined in accordance with the Sponsor. The following may be checked, according to the monitoring plan:

- informed consent;
- compliance with the study protocol and the procedures defined therein;
- quality of the data collected in the case report: accuracy, missing data, consistency of data with source documents;
- management of the experimental procedure;
- reporting of serious adverse events.

Each visit will be the subject of a monitoring report kept by the sponsor and, if necessary, a list of actions to be implemented sent to the investigator of the center visited and to the research coordination structure. Furthermore, the investigators agree to accept quality assurance audits by persons mandated by the sponsor and inspections by the Competent Authorities. All data, documents and reports may be subject to regulatory audits and inspections without prejudice to medical confidentiality.

13 ETHICAL CONSIDERATIONS

13.1 Competent Authorities

The protocol, the patient information sheet and the consent form for the study will be submitted to the Ethics Committee for approval. The study will start only after obtaining a favorable opinion from the competent Ethics Committee from all the study sites.

13.2 Substantial changes

In the event of substantial modification of the protocol by the investigator, it must be approved by the Sponsor. Prior to its implementation, the Sponsor must obtain a favorable opinion from the Ethics Committee. A new consent from the persons participating in the research will be obtained if necessary.

13.3 Patient information and written consent form, including emergency consent procedure (deferred consent)

Patients eligible for enrolment will be identified at admission, once routine neuroimaging has been completed. All patients with AIS due to left or right M1 occlusion will be screened for participation. Inclusion and exclusion criteria will be assessed based on information available at admission.

The investigator’s decision to enroll a patient must be based on an appropriate benefit–risk balance. The patient, or his/her legally authorized representative, will be fully and fairly informed, in understandable terms, about the objectives and constraints of the study, the possible risks, the required monitoring and safety measures, and the right to refuse participation or to withdraw at any time. This information is provided in an information and consent form, and written informed consent will be obtained by the investigator prior to inclusion.

If the patient is unable to provide informed consent and no legally authorized representative is available at the time of enrollment, an emergency consent procedure (EU 536/2014, section 36; 2025 AIFA guidelines) will be applied. Considering the AIS emergency situation, the time-dependent nature of the experimental treatment, and the favorable safety profile of HDT15, the investigator will inform the patient’s next of kin, if available, about the study. In such cases, the investigator documents in the medical record that the emergency consent procedure has been applied and signs the relevant documentation. The patient or his/her legal representative must then provide full written informed consent as soon as the patient’s clinical condition allows it (deferred consent). Emergency consent must always be documented before performing any clinical or paraclinical examination required by the study.

In all cases, the patient’s assent will be explored. If the investigator has reason to believe that the patient would refuse the procedure, the treatment will not be administered.
If the patient, or the legally authorized representative (or the next of kin in case of emergency consent), decides to withdraw from the trial, the patient will be discontinued from the study.

In all cases, the investigator must record the progress of the inclusion in the medical file, and written informed consent must be obtained as soon as the patient’s clinical condition allows it.

13.4 Declaration of conformity

The sponsor and the investigator guarantee that this research will be conducted:

- in accordance with the protocol;
- in accordance with current international good clinical practice rules;
- in accordance with the legislative and regulatory provisions currently in force in Italy and internationally.

13.5 Exclusion period

Patients participating in this study cannot simultaneously participate in another interventional research protocol. No exclusion period is needed after the end of the trial.

13.6 Compensation for subjects and/or registration in the national file of persons undergoing intervention research on human subjects of 1°.

There is no intention to compensate patients who agree to participate in the study.

14 DATA MANAGEMENT AND RETENTION

14.1 Case report form

The case report form (CRF) will only contain the data necessary for the analyses for publication. The other data relating to the patient and necessary for their follow-up outside the study will be collected in their medical file.

The study data will be collected in an electronic case report (eCRF). This eCRF, specific to the study, will be developed by a data manager from the University of Milano-Bicocca.

The eCRF will only include data necessary for the protocol and scientific publication. Other patient data necessary for follow-up outside of this study will be collected in the patient's medical record. The study data will be computerised in a coded manner, in accordance with the European Data Protection Act. Study subjects will be identified by their unique study inclusion number (study code). A list of research participants' identification will be kept in the investigator's site file. The data must be completed, as they are collected, by the authorised persons (investigator and persons appearing on the delegation of tasks) and with their own identifiers. Filling in the case report via the Internet allows the coordination center and the study sponsor to view the data quickly and remotely. When data is entered, it is immediately checked for consistency. The person in charge of filling in the data must validate and justify any change in value in the eCRF. Entries and modifications are subject to an audit trail. The investigator is responsible for the accuracy, quality and relevance of all data entered. As such, each page of the patient CRF must be dated and electronically signed by the investigator, signifying their agreement with and responsibility for the data collected.

14.2 Data management

The simple entry of data will be carried out by the the investigators in an eCRF. The data will be validated in conformity with the data management plan defined jointly by the coordinating investigator, the methodologist, the data manager and the statistician. The freezing/unfreezing process for the data will be carried out in conformity with the procedure put in place in the study coordination center.
All of the data is stored in a datacenter with a back-up and kept for 15 years.

14.3 Archiving

The following documents will be archived by the name of the study under the responsibility of the coordinating investigator and associated investigators at each center for a period of 15 years:

- protocol and annexes, possible amendments;
- original signed information forms and consents;
- individual data (authenticated copies of raw data);
- follow-up documents and correspondence relating to the research.

The Sponsor is also responsible for organising the storage of the statistical analyses and the final report of the study for the regulatory archiving period. No removal or destruction will be carried out without the agreement of the sponsor. At the end of the 15-year period, the

sponsor will be consulted for destruction. All data, documents and reports may be subject to audit or inspection.

15 FUNDING AND INSURANCE

15.1 Study funding

Funding is provided by the Italian Minitry of University and Research (MUR), grant PRIN 2022 Prot. 2022LNL3H3 (CUP: H53D23005490006).

15.2 Insurance

The sponsor will take out insurance for the entire duration of the study to cover its own civil liability and that of any doctor involved in the study. The sponsor will also ensure full compensation for the harmful consequences of the research for the person who takes part in the study and their beneficiaries, unless it can be proved that the damage is not attributable to its fault or to that of any other party involved, without the possibility of invoking the actions of a third party or the voluntary withdrawal of the person who initially agreed to take part in the research.

16 RULES FOR PUBLICATION

Scientific papers and reports related to this study will be produced under the responsibility of the principal investigator of the study with the agreement of the associate investigators. The co-authors of the report and publications will be the investigators and clinicians involved, in proportion to their contribution to the study, as well as the biostatistician and the associated researchers. If a meta-analysis is carried out and contracted with the French partner (Prof. Tae-Hee Cho, Hospices Civils de Lyon, France), the authors' position will be discussed according to the contribution of each partner. The study will be registered on an open access clinical trial registry (clinicaltrials.gov) prior to the inclusion of the first patient.

17 REFERENCES

1. Powers WJ, Rabinstein AA, Ackerson T, Adeoye OM, Bambakidis NC, Becker K et al. Guidelines for the Early Management of Patients With Acute Ischemic Stroke: 2019 Update to the 2018 Guidelines for the Early Management of Acute Ischemic Stroke: A Guideline for Healthcare Professionals From the American Heart Association/American Stroke Association. Stroke. 2019;50:e344-e418.

2. Goyal M, Menon BK, van Zwam WH, Dippel DWJ, Mitchell PJ, Demchuk AM et al. Endovascular thrombectomy after large-vessel ischaemic stroke: a meta-analysis of individual patient data from five randomised trials. Lancet. 2016;387:1723-1731.

3. Shuaib A, Bornstein NM, Diener H-C, Dillon W, Fisher M, Hammer MD et al. Partial aortic occlusion for cerebral perfusion augmentation: safety and efficacy of NeuroFlo in Acute Ischemic Stroke trial. Stroke. 2011;42:1680-1690.

4. Guluma KZ, Liebeskind DS, Raman R, Rapp KS, Ernstrom KB, Alexandrov AV et al. Feasibility and Safety of Using External Counterpulsation to Augment Cerebral Blood Flow in Acute Ischemic Stroke-The Counterpulsation to Upgrade Forward Flow in Stroke (CUFFS) Trial. J Stroke Cerebrovasc Dis. 2015;24:2596-2604.

5. Bornstein NM, Saver JL, Diener HC, Gorelick PB, Shuaib A, Solberg Y et al. An injectable implant to stimulate the sphenopalatine ganglion for treatment of acute ischaemic stroke up to 24 h from onset (ImpACT-24B): an international, randomised, double-blind, sham-controlled, pivotal trial. Lancet. 2019;394:219-229.

6. Ginsberg MD, Palesch YY, Hill MD, Martin RH, Moy CS, Barsan WG et al. High-dose albumin treatment for acute ischaemic stroke (ALIAS) part 2: a randomised, double-blind, phase 3, placebo-controlled trial. Lancet Neurol. 2013

7. Bang OY, Chung JW, Kim SK, Kim SJ, Lee MJ, Hwang J et al. Therapeutic-induced hypertension in patients with noncardioembolic acute stroke. Neurology. 2019;93:e1955-e1963.

8. Beretta S, Versace A, Carone D, Riva M, Dell'Era V, Cuccione E et al. Cerebral collateral therapeutics in acute ischemic stroke: A randomised preclinical trial of four modulation strategies. J Cereb Blood Flow Metab. 2017;37:3344-3354.

9. Diamanti S, Mariani J, Versace A, Riva M, Cuccione E, Cai R et al. Head down tilt 15° to preserve salvageable brain tissue in acute ischemic stroke: A preclinical pooled analysis, with focus on cerebral hemodynamics. Eur J Neurosci. 2022

10. Anderson CS, Arima H, Lavados P, Billot L, Hackett ML, Olavarría VV et al. Cluster-Randomised, Crossover Trial of Head Positioning in Acute Stroke. N Engl J Med. 2017;376:2437-2447.

11. Gauthier A, Gérardin P, Renou P, Sagnier S, Debruxelles S, Poli M et al. Trendelenburg Positioning in Large Vessel Ischaemic Stroke: A Pre-Post Observational Study Using Propensity Score Matching. Cerebrovasc Dis. 2018;46:24-32.

12. Higashida RT, Furlan AJ, Roberts H, Tomsick T, Connors B, Barr J et al. Trial design and reporting standards for intra-arterial cerebral thrombolysis for acute ischemic stroke. Stroke. 2003;34:e109-37.

13. Liebeskind DS, Tomsick TA, Foster LD, Yeatts SD, Carrozzella J, Demchuk AM et al. Collaterals at angiography and outcomes in the Interventional Management of Stroke (IMS) III trial. Stroke. 2014;45:759-764.

14. Tan IY, Demchuk AM, Hopyan J, et al. CT angiography clot burden score and collateral score: correlation with clinical and radiologic outcomes in acute middle cerebral artery infarct. AJNR Am J Neuroradiol. 2009;30(3):525-531. doi:10.3174/ajnr.A1408
